# Dynamic responses and implications to coastal wetlands and the surrounding regions under sea level rise

**DOI:** 10.1371/journal.pone.0205176

**Published:** 2018-10-12

**Authors:** Karim Alizad, Scott C. Hagen, Stephen C. Medeiros, Matthew V. Bilskie, James T. Morris, Len Balthis, Christine A. Buckel

**Affiliations:** 1 Louisiana State University, Center for Coastal Resiliency, Baton Rouge, Los Angeles, United States of America; 2 Louisiana State University, Center for Coastal Resiliency, Center for Computation & Technology, Department of Civil, and Environmental Engineering, Baton Rouge, Los Angeles, United States of America; 3 University of Central Florida, Department of Civil, Environmental, and Construction Engineering, Orlando, Florida, United States of America; 4 Department of Biological Sciences and Belle W. Baruch Institute, University of South Carolina, Columbia, South Carolina, United States of America; 5 National Oceanic and Atmospheric Administration, National Ocean Service, National Centers for Coastal Ocean Science, Silver Spring, Maryland, United States of America; Universidade de Aveiro, PORTUGAL

## Abstract

Two distinct microtidal estuarine systems were assessed to advance the understanding of the coastal dynamics of sea level rise in salt marshes. A coupled hydrodynamic-marsh model (Hydro-MEM) was applied to both a marine-dominated (Grand Bay, Mississippi) and a mixed fluvial/marine (Weeks Bay, Alabama) system to compute marsh productivity, marsh migration, and potential tidal inundation from the year 2000 to 2100 under four sea level rise scenarios. Characteristics of the estuaries such as geometry, sediment availability, and topography, were compared to understand their role in the dynamic response to sea level rise. The results show that the low sea level rise scenario (20 cm) approximately doubled high-productivity marsh coverage in the marine-dominated estuary by the year 2100 due to an equilibrium between the rates of sea level rise and marsh platform accretion. Under intermediate-low sea level rise (50 cm), high-productivity marsh coverage in the year 2100 increased (doubled in the marine-dominated estuary and a seven-fold increase in the mixed estuary) by expanding into higher lands followed by the creation of interior ponds. The results also indicate that marine-dominated estuaries are vulnerable to collapse as a result of low, relatively uniform topography and lack of sediment sources, whereas mixed estuaries are able to expand due to higher elevations and sediment inputs. The results from the higher sea level rise scenarios (the intermediate-high (120 cm) and high (200 cm)) showed expansion of the bays along with marsh migration to higher land, producing a five-fold increase in wetland coverage for the mixed estuary and virtually no net change for the marine-dominated estuary. Additionally, hurricane storm surge simulations showed that under higher sea level rise scenarios, the marine-dominated estuary demonstrated weaker peak stage attenuation indicating that the marsh’s ability to dissipate storm surge is sensitive to productivity changes and bay expansion / marsh loss.

## Introduction

Coastal wetlands are valuable ecosystems due to their role in coastal protection, water purification, carbon sequestration, erosion control, and as a habitat and food source for many species [1–3]. The ecosystems in microtidal regions, such as the Mediterranean Sea and the northern Gulf of Mexico (NGOM), are more susceptible to marsh productivity and coverage losses due to sea level rise (SLR) [4–9]. Their vulnerability to SLR is typically due to the excessive inundation of low marsh regions in terms of both time and depth along with an insufficient allochthonous sediment supply to the marsh platform that hampers its ability to accrete and keep pace with rising water levels [8,9]. Estuarine systems generally respond to SLR by accumulating or releasing sediments in an effort to find a vertical equilibrium position within the tidal frame. The local responses are dynamically driven by hydrodynamic (tidal and river) and geometric conditions [10–12] that also serve to characterize estuaries as fluvial, marine-dominated, or fluvial/marine (henceforth referred to as mixed). Marine-dominated estuaries are characterized by the absence of fluvial inflow, uniform water levels in the creeks and bays, and a simple creek network. Conversely, fluvial and mixed estuaries receive river flow, including sediment, and contain more complex creek networks and estuarine geometry due to tidal and river flow interactions [13–15]. Coastal wetlands lie at the nexus of the hydrodynamic, geometric and ecologic regimes of an estuary. Therefore, the approach to assessing the impacts of SLR presented here focuses on understanding the dynamic response of coastal salt marsh productivity under SLR scenarios. These assessments can help coastal resource managers choose suitable protection and restoration planning pathways [16].

SLR can effectively change the hydrodynamics in estuaries [17–19], which are inherently dynamic due to the nonlinear hydrodynamic and morphological response of the estuaries to SLR [20]. In order to assess salt marsh response to SLR, it is important to use models that include the interactions between local hydrodynamics and the salt marsh system [21]. Several integrated models have been developed and applied in various regions to study SLR impacts on coastal wetlands [22–26]. Due to the small tide range and acute sensitivity of microtidal marsh systems to their hydroperiod, it is necessary to use a model that resolves intricate water level variations in the tidal creeks and rivers. The Hydro-MEM model [25] was selected for this study because it incorporates the dynamics of tides and marsh biomass productivity under SLR [27] by coupling hydrodynamic and parametric marsh models. Hydro-MEM incorporates the accelerating rate of SLR using a decadal coupling framework to include the interaction between the marsh and the estuary hydrodynamics by adjusting both the marsh platform elevation (from accretion or land loss) and representative bottom friction (from changes in vegetation density) at each coupling time step [25,28]. The Hydro-MEM model requires an accurate topographic elevation model of the marsh platform [29], Marsh Equilibrium Model (MEM) experimental parameters [30], SLR rate projections, initial spatially-distributed bottom friction parameters (Manning’s *n*) [31], and a high-resolution hydrodynamic model [28]. The effort required for this integrated and complex modeling approach is warranted by the importance of marsh systems to both global and local economies.

Research has shown that marshes play an essential role in protecting shorelines and other coastal assets by increasing bottom friction and dissipating waves [32–37]. Specifically, their along- and cross-shore extents, productivity (biomass density), and bathymetry influence their ability to attenuate storm surge and waves [3,38,39]. Studies have shown that under moderate and high rates of SLR, marshes are likely to be entirely inundated, lose productivity, and ultimately transition to open water [26,40]. These changes to the biogeomorphology of the system result in variable hydrodynamic changes [24,25,28,41]. In response to these new local hydrodynamics, the marsh landscape adapts to changes in sediment supply and salinity, the creation of ponds, and new wave propagation patterns which often combine to widen back bays and create new or widen existing tidal creeks [42–45]. In addition, marsh systems often respond to SLR by migrating to higher lands if they are not obstructed by development or urban infrastructure [28,46,47]. These potential regions for marsh migration in urban, agricultural, or forested areas must be assessed and mapped using marsh evolution models and preserved for restoration purposes [48,49].

Rates of marsh migration on gently sloping coastal planes are more sensitive to accelerated SLR than rates of edge erosion [47,50]. Previous studies have shown that if marshes are allowed to freely migrate upland, the net loss of marsh coverage could be minimal or in some cases even increase[49,50]. Facilitating this free migration may require removing barriers (*e*.*g*. roadways) and restoring developed land back to open space with appropriate topography. These changes in the landscape play an important role when attempting to accurately project future ecosystem services such as protection of coastal assets through storm surge dissipation. However, limited studies have discussed the salt marsh role on storm surge attenuation due to the complex physics and numerous physical parameters that must be derived or assumed [35]. Observations and recorded data have resulted in a heuristic approximation of 1 meter peak storm surge attenuation per 14.5 to 20 km of marsh, although the influential physical factors such as marsh system, storm track, and topography were not investigated [51,52]. Examining the findings from past hurricanes hindcasted with advanced computer models indicates the importance of including detailed characteristics of the system including topography, bathymetry, and wetland parameters in addition to storm size, track, speed, and intensity to storm surge attenuation in different marsh systems [3,38,39,53,54]. In addition, published research demonstrates that while marsh systems play a critical role in protecting shorelines from storm surge, there is no general relationship between surge attenuation and marsh coverage due to the complex physical processes at work [3,35,39,55]. Few studies have focused SLR impact to both marsh ecologic health and its ability to buffer storm surge, therefore there is a need to synthesize these responses. This was done by applying the dynamic effects of marsh productivity, bottom friction, and topography and bathymetry variations, as well as storm surge amplification under different SLR scenarios.

This manuscript presents a major part of a comprehensive study of the ecological effects of SLR. The main objective of this study was to assess and compare the dynamic response of two distinct microtidal estuaries in Grand Bay, MS (marine-dominated) and Weeks Bay, AL (mixed) to SLR and advance the findings of a study of the fluvial-dominated microtidal system in Apalachicola, FL [28]. These dynamic responses were assessed by developing a model to project and map the changes in marsh productivity and potential upland migration. In addition, the changes in storm surge attenuation resulting from SLR-induced marsh system changes were quantified. This analysis incorporated marsh platform accretion, upland migration, bay expansion, and productivity changes into the model to measure their effect on storm surge attenuation. The knowledge gained from this study builds upon previous efforts [28] to understand the coastal dynamics of sea level rise in diverse estuarine systems.

## Study area

**Fig 1:**
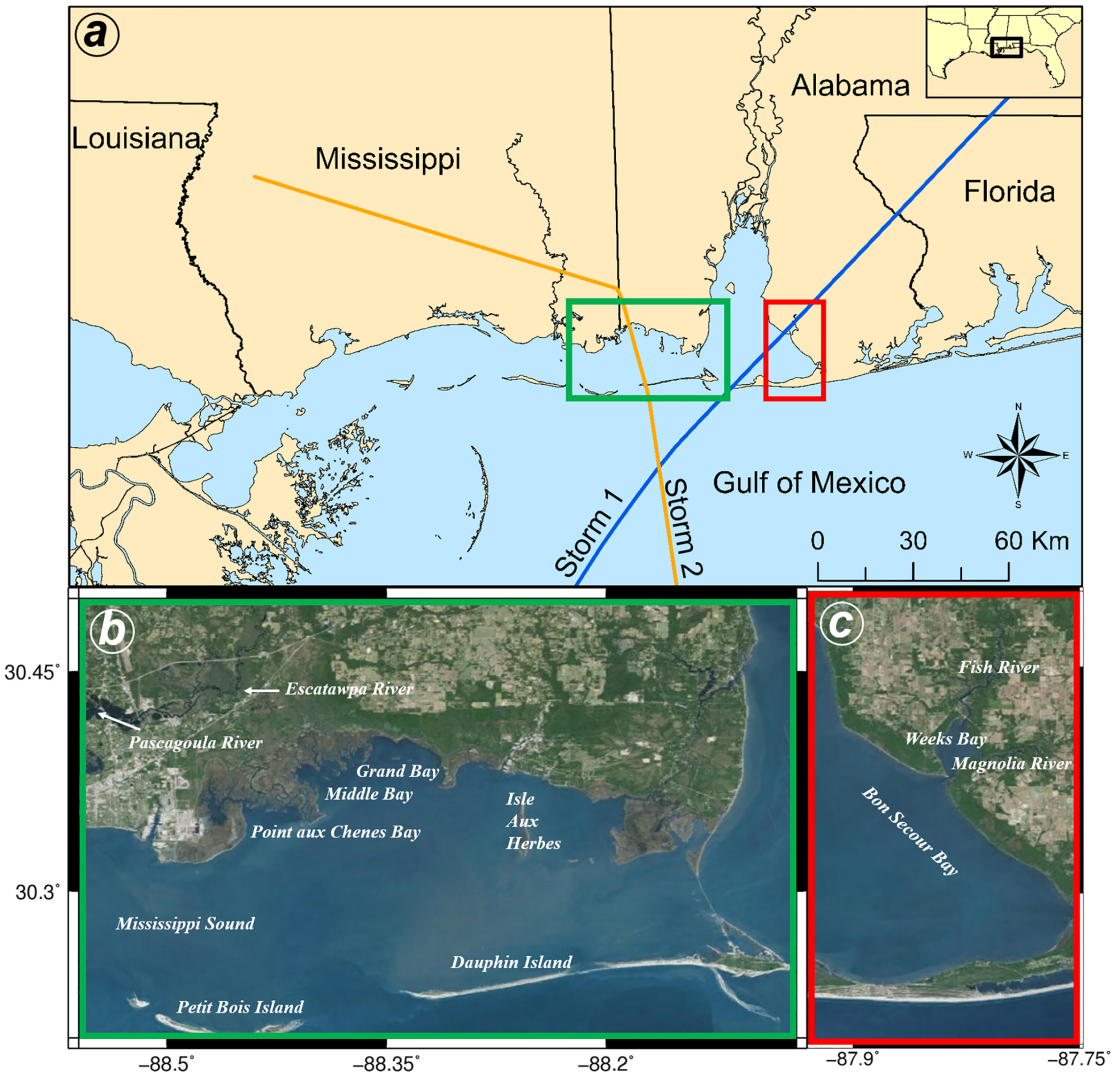
Study area and location of the Grand Bay estuary (b) and Weeks Bay estuary (c) in the NGOM as well as two synthetic storm tracks (a). The basemaps in this figure are screen captures of World Imagery map in ArcGIS [64].

Grand Bay and Weeks Bay estuaries are categorized as a marine-dominated estuary and a mixed estuary, respectively. Grand Bay is one of the largest intact coastal systems in Mississippi, located near the southern extent of the Mississippi and Alabama state line (Fig 1b), and consists of several shallow bays (0.5 m to 3 m in Point aux Chenes Bay) that are effective in damping wave energy [56–58]. Grand Bay estuary includes 41 km^2^ of tidal wetlands and swamps dominated by *Juncus romerianus* and *Spartina alterniflora* [59]. The marsh serves as a nursery and habitat for commercially harvested species such as shrimp, crabs, and oysters [57,60]. Historically, this marsh system has been prone to erosion at a rate of 1.7 m/yr primarily due to SLR [61,62]. The main factors contributing to this erosion are (1) the Escatawpa River diversion in 1848, which was the main estuary’s sediment source, and (2) the Dauphin Island breaching caused by a hurricane in the late 1700s that allowed the propagation of larger waves and tidal flows from the Gulf of Mexico [58,60,63]. In addition, waves currently break on the marsh platform edge causing the loss of large masses of marsh when the water level is low. When the water level is high, waves break on top of the marsh platform and undermine the marsh, opening narrow channels that permeate the marsh and accelerate erosion [60].

The Weeks Bay estuary, located along the eastern shore of Mobile Bay in Baldwin County, AL is categorized as a mixed estuary (Fig 1). Weeks Bay consists of bottomland hardwood forest followed by intertidal salt marsh habitat for animals and nurseries for commercially harvested species such as shrimp, blue crab, shellfish, bay anchovy, and others [65,66]. This estuary is principally affected by the fresh water inflow from the Magnolia River (25%), the Fish River (73%), and some smaller channels (2%) with a combined annual average discharge of 5 cubic meters per second [67]. Mobile Bay is the estuary’s coastal ocean salt source. Sediment is transported to the bay by the Fish River during winter and spring as a result of overland flow from rainfall events but is minimal during summer and fall when the discharge is typically low [65]. Three dominant marsh species are *J*. *romerianus* and *S*. *alterniflora* in the higher salinity regions near the mouth of the bay and *Spartina cynosuroides* in the brackish region at the head of the bay. In the calm waters near the sheltered shorelines, submerged aquatic vegetation (SAV) is dominant. The future of the Weeks Bay ecosystem is threatened by urbanization, which is the most significant change in the Weeks Bay watershed in recent decades [66]. Also, as a result of SLR, some marshes in the Weeks Bay have already converted to open water [68] highlighting the importance and urgency of this study.

## Methods

**Fig 2:**
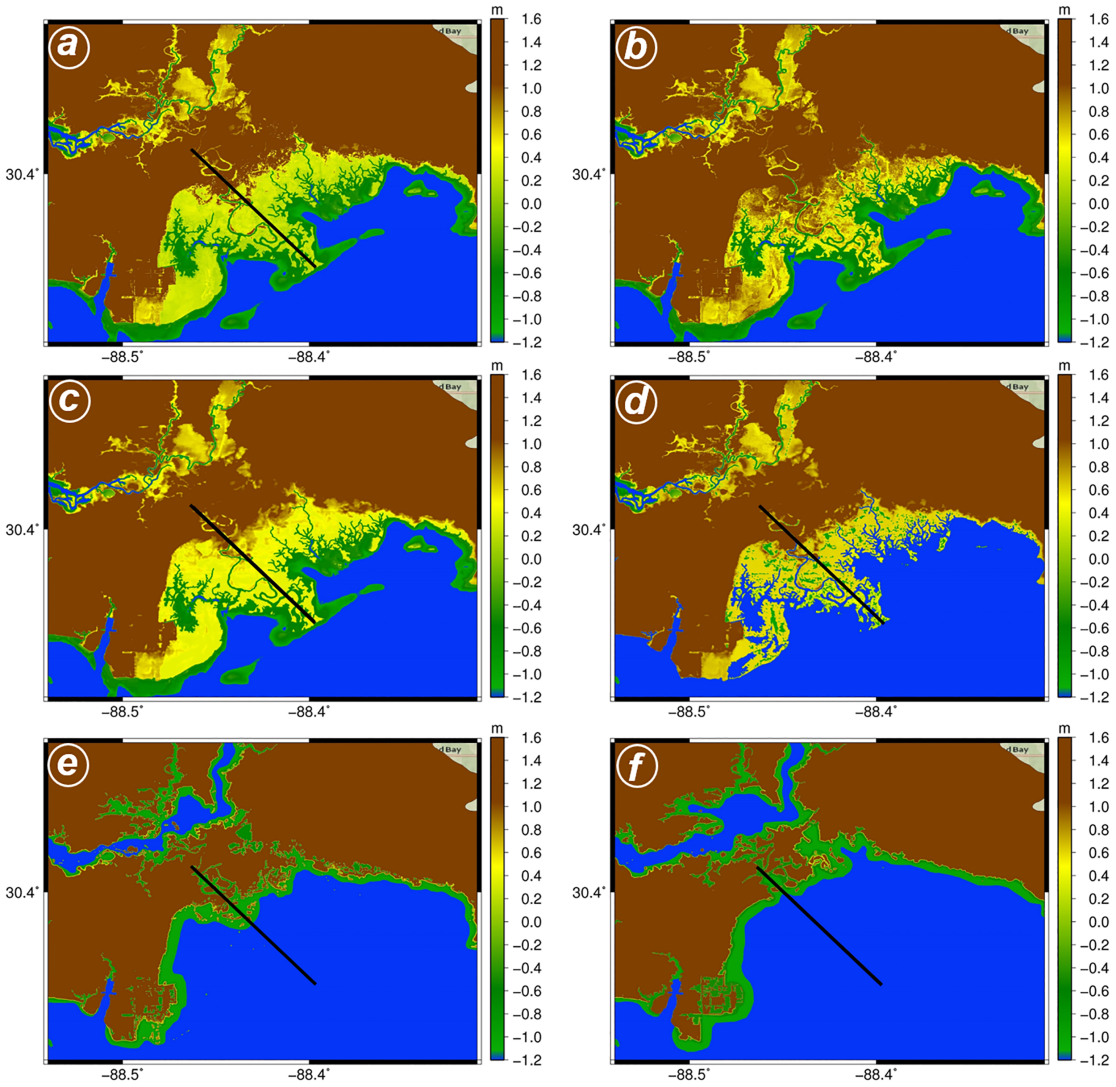
Grand Bay estuary topography for the current condition and under different SLR scenarios. The adjusted topography for current condition (c. 2000) is shown in (a). The unadjusted topography used for all scenarios is shown in (b). The maps from c-f shows the updated adjusted landscape (dynamic landscape—topographic change based on salt marsh platform accretion and bathymetric change for the expanded bay were applied) in the year 2100 under the low (c), intermediate-low (d), intermediate-high (e), and high (f) SLR scenarios, respectively. The blue and green (shallow water) colors show wet area, yellow is low land and brown demonstrates higher lands and the black line shows the transect used for assessment.

The Hydro-MEM model [25] was used to compute marsh productivity for present and future conditions under four SLR projections. The inputs to the Hydro-MEM model include tidal forcings, river inflow, present day marsh platform topography (Fig 2a), and Marsh Equilibrium Model (MEM) constants. The marsh productivity results were validated using field data and employed to generate marsh migration-possibility maps. In addition, marsh productivity results were integrated in a hurricane storm surge model to investigate changes in storm surge attenuation within Grand Bay.

### Hydro-MEM model

Coastal wetland response to SLR was assessed using the integrated Hydro-MEM model [25] consisting of coupled hydrodynamic and parametric marsh (MEM) models. It was developed to model the interconnection between the major biogeophysical components of marsh systems by capturing feedback processes in a time-stepping framework. The time step approach served to incorporate the rate of SLR by capturing the two-way feedback between the salt marsh system (vegetation and topography) and hydrodynamics after each time step. Additionally, the model implemented the dynamics of SLR by using a hydrodynamic model to provide input to the MEM in the form of tidal parameters. The marsh platform elevation and Manning’s *n* values were updated using the biomass density and accretion results from MEM and then used as input to the hydrodynamic model. Once the model reached the target time, the simulation stopped and generated results [25].

The MEM component of the model used mean high water (MHW), topography, and experimental parameters to calculate biomass density at each computational node. The MEM utilized a parabolic curve that was a function of a relative depth (*D*), which is defined by subtracting the topographic elevation from the computed MHW elevation. The parabolic curve determined biomass density *(B)* as a function of this relative depth [30] as follows:

$$B=aD+bD^{2}+c$$ (1)

where *a*, *b*, and *c* are unique, experimentally derived parameters for each estuary. In this study, the parameters were obtained from field bio-assay experiments [28,30,69] in both Grand Bay and Weeks Bay. The fit of a simple parabolic curve to bioassay data can be quite good (r^2^ = 0.73, p = .0001) [69]. However, the variability in field data at a given elevation is typically high and it was necessary to adjust the parameters to account for asymmetries. The curve for Grand Bay was divided into sub-optimal (left) and super-optimal (right) branches that met at the parabola apex. The left and right parameters for Grand Bay [70] were *a*_*l*_ = 32 *gm*^−3^ *yr*^−1^, *b*_*l*_ = 32 *gm*^−4^ *yr*^−1^, *c*_*l*_ = 1920 *gm*^−2^ *yr*^−1^ and *a*_*r*_ = 6.61 *gm*^−3^ *yr*^−1^, *b*_*r*_ = −0.661 *gm*^−4^ *yr*^−1^, *c*_*r*_ = 1983 *gm*^−2^ *yr*^−1^, respectively. The biomass density curve for Weeks Bay had no asymmetry and was thus defined using the parameters *a* = 73.8 *gm*^−3^ *yr*^−1^, *b*_*l*_ = −1.14 *gm*^−4^ *yr*^−1^, *c* = 1587.1 *gm*^−2^ *yr*^−1^. Additionally, the MEM element of the model calculated the organic and inorganic accretion rates on the marsh platform using an accretion rate formula that incorporated the parameters for inorganic sediment load (*q*) and organic and inorganic accumulation generated by decomposing vegetation (*k*) [30]. Based on this relationship, salt marsh platform accretion depended on the productivity of the marsh, the amount of sediment input, and the water level during the high tide [30,71–75]. It also depended on the coupling time step (*dt*) at which the elevation and bottom friction inputs were updated in the hydrodynamic model.

### Hydrodynamic model

The hydrodynamic model component of Hydro-MEM was the two-dimensional, depth-integrated ADvanced CIRCulation (ADCIRC) model that solves the shallow water equations over an unstructured finite element mesh [76,77]. The mesh for this study had a resolution or horizontal node spacing of 15 m, on average, in the marsh regions in the Grand Bay and Weeks Bay estuaries. This high resolution mesh for Grand Bay and Weeks Bay was fused to an existing mesh developed by Bilskie et al. (78). The mesh spans the western north Atlantic from the open ocean boundary at the 60 degree west meridian through the Atlantic Ocean, Gulf of Mexico, and the Caribbean Sea and contains 1,095,214 nodes. This newly combined mesh was developed with consideration of numerical stability in cyclical floodplain wetting [79] and the techniques to capture tidal flow variations within the marsh system [28].

Inputs to the model consisted of Manning’s *n*, initial water level (with SLR), and topography. The initial values for Manning’s *n* were derived using the National Land Cover Database (NLCD 2001) [31] along with *in situ* observations [80]. Over the course of the model runs, the Manning’s *n* values were updated based on the biomass density level of low (0.035), medium (0.05), and high (0.07) or reclassified as open water (0.022) [28,81]. Additionally, the four SLR projections for the year 2100 used in Hydro-MEM were low (0.2 m), intermediate-low (0.5 m), intermediate-high (1.2 m) and high (2.0 m) [82]. The coupling time step for low and intermediate-low scenarios was 10 years, and it was 5 years for the intermediate-high and high scenarios [25]. Therefore, the SLR at each time step varied based on the individual SLR projections. In addition, the hydrodynamic model was forced by seven principal harmonic tidal constituents (M_2_, S_2_, N_2_, K_1_, O_1_, K_2_, and Q_1_) along the open ocean boundary and river inflow at the Fish River and Magnolia River boundaries. No flow boundary conditions were applied along the coastline and upland extent of the coastal floodplains. The river discharge boundary conditions were calculated as the mean discharge from 44 years of record for the Fish River (http://waterdata.usgs.gov/usa/nwis/uv?site_no=02378500) and 15 years of record for Magnolia River (http://waterdata.usgs.gov/nwis/uv?site_no=02378300). As of February 2016, these values were 3.18 and 1.11 cubic meters per second, respectively. The hydrodynamic model forcings were ramped by two tangential functions: one for the tidal constituents and the other for river inflows. To capture the nonlinearities and dynamic effects induced by geometry and topography, the output of the model in the form of harmonic tidal constituents were resynthesized and analyzed to produce MHW in the rivers, creeks, and marsh system. This model has been extensively validated and applied in several studies [78,83–85].

### Topography adjustment

The hydrodynamic model elevation was interpolated onto the mesh using an existing digital elevation model (DEM) (Fig 2b) developed by Bilskie et al. [86], [87] as a basis and incorporating a necessary adjustment to the marsh platform [28] due to the high bias of the lidar-derived elevations in salt marshes [29]. Since this marsh is biologically similar to Apalachicola, FL (*J*. *romerianus* dominated, *Spartina* fringes, and similar above-ground biomass density), the elevation adjustment involved constraining the marsh platform elevations so that areas of similar productivity were vertically positioned relative to the tidal frame similar to Apalachicola, while preserving the natural micro-topographic variability.

In Grand Bay, the local tidal datums for MHW, mean sea level (MSL) and mean low water (MLW) are 0.273 m, 0.053 m and -0.144 m, respectively, referenced to NAVD88. This results in an upper tide range (MSL to MHW) of 0.220 m and an upper midpoint of 0.160 m. In Apalachicola, the productive areas of the marsh were also in the upper portion of the tidal frame so this was used to guide the elevation adjustment in Grand Bay. If the unadjusted DEM elevation was 0.160 m or lower, no further adjustment was made. This threshold was established coincident with the midpoint of the upper portion of the tidal frame; if the unadjusted DEM yielded an elevation of 0.160 m or lower, it is a signature of low lidar DEM bias and should not be adjusted. In practice, adjusting raw elevations lower than the midpoint of the upper portion of the tidal frame flattened the microtopography of the small tidal creeks and unrealistically altered the inundation pattern.

For unadjusted DEM elevations higher than 0.160 m, the following equation was used to constrain the elevations to a realistic range based on the vertical distribution of similar vegetation in Apalachicola.

$$z_{adj}=\frac{\left(u-l)(z_{raw}-z_{\text{min}}\right)}{\left(z_{\text{max}}-z_{\text{min}}\right)}+l$$ (2)

where *z*_*adj*_ is the adjusted elevation; *u* and *l* are the desired upper and lower limits of the adjusted elevations, respectively; and *z*_max_ and *z*_min_ are the actual maximum and minimum raw elevations of the DEM cells to be adjusted in the domain. For Grand Bay, some manual tuning of these values was necessary to avoid unrealistic uniformity in the adjusted DEM. *u* and *l* were set to 0.493 m and 0.160 m, respectively. An elevation of 0.160 m was set as the floor of the constrained range to coincide with the midpoint of the upper portion of the tidal range. An elevation of 0.493 m was set as the ceiling of the constrained range to provide enough numerical space for the variability to propagate to the adjusted DEM. This ceiling value was computed by adding the span of the upper tide range (0.220 m) to the MHW elevation. *z*_max_ and *z*_min_ were set to 1.20 and 0.160 m, respectively. The value of 1.20 m was selected because it represented a natural inflection point in the distribution of elevations and including higher elevations, that only occur rarely across the marsh and are possibly outliers, would restrict the adjustment to an unrealistically narrow range. The adjusted and unadjusted DEMs are shown in the Fig 2a and 2b, respectively.

### Marsh coverage validation

**Fig 3:**
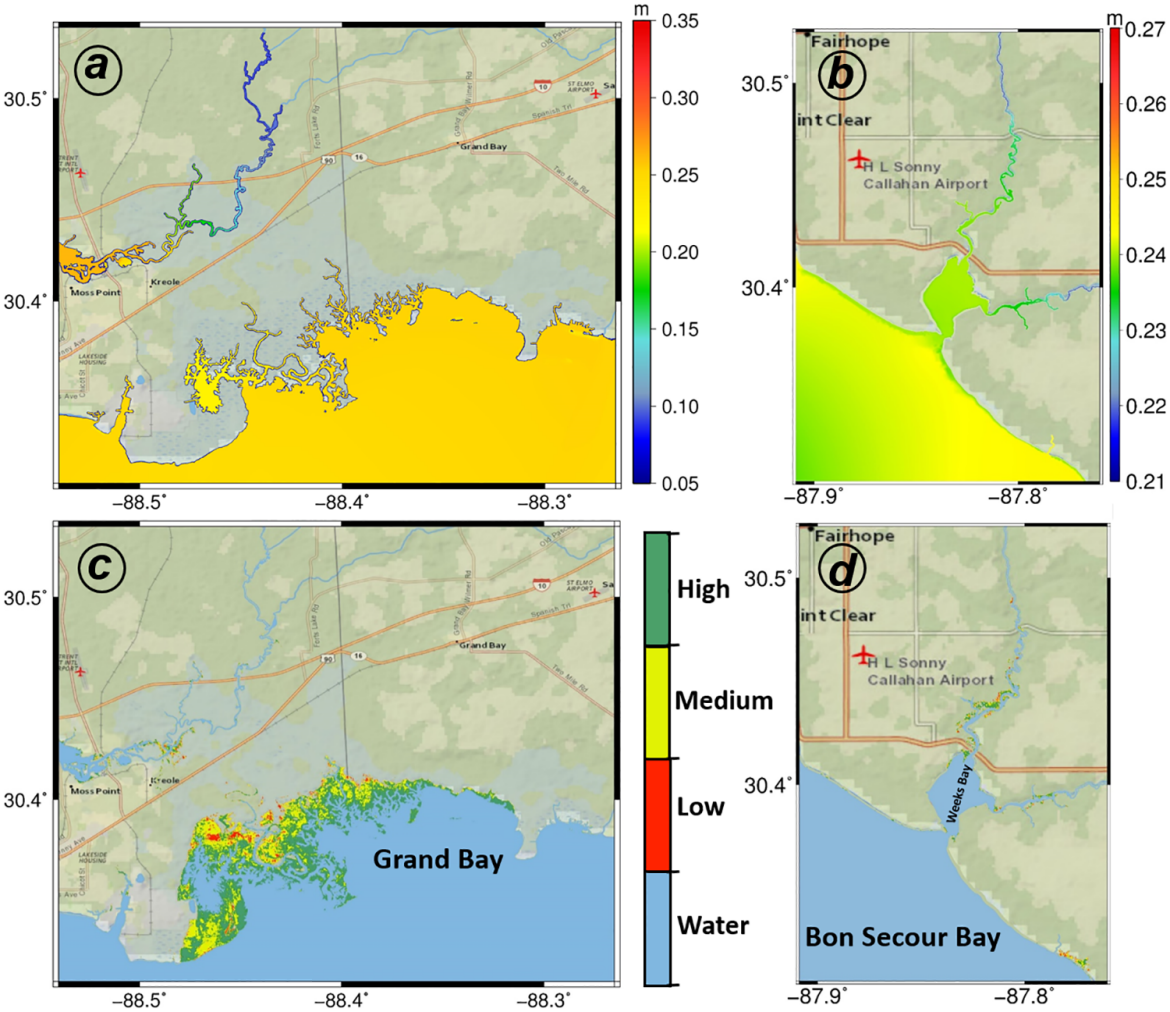
MHW and marsh productivity maps in Grand Bay and Weeks Bay for current condition (c. 2000). The top row shows MHW for the current sea level in Grand Bay (a) and Weeks Bay (b) where warmer colors represent higher water levels. The bottom row demonstrates salt marsh productivity for the Grand Bay (c) and Weeks Bay (d) estuary and blue, red, yellow, and green colors represent water, and low, medium, and high productivity marsh, respectively. The basemaps in this figure are screen captures of National Geographic World map in ArcGIS [95].

To investigate the potential for salt marsh migration and its effect on storm surge, the model boundary in Grand Bay and Weeks Bay was extended beyond the National Estuarine Research Reserve (NERR) boundary. The model boundary in Grand Bay was extended west from the Pascagoula River and east to Isle Aux Herbes, and it was extended north to include portions of the Escatawpa River. In Weeks Bay, the model included Bon Secour Bay and was extended from the east to the cross of Magnolia River and US Highway 98 and from the north to State Highway 104 (Figs 1 and 3). However, the data for validation was located within the boundary of Grand Bay National Estuarine Research Reserve (GBNERR) and it includes most of the salt marsh area. The Hydro-MEM model results for Grand Bay under the intermediate-low 2020 SLR scenario were compared against two separate high-resolution land cover sources that explicitly identify marsh habitats. The reason for selecting the intermediate-low 2020 SLR scenario is that it was the closest result in time to the 2017 data from GBNERR and National Wetlands Inventory (NWI) [88,89] used for validation. In addition, this SLR scenario (5 cm rise from 2000–2020) was in line with the lower end (5.76 cm) of the observed mean sea level trend of 3.50 mm/year +/- 0.62 mm/yr (95% confidence limits) based on monthly mean sea level data from 1966 to 2016 at Dauphin Island, Alabama, USA [90].

Habitat classifications [88] were obtained from the GBNERR as a 1.2-m raster and resampled to 10 m resolution to match that of the Hydro-MEM model output raster. In addition, wetlands classifications for coastal Mississippi [89] were downloaded from the NWI as shapefiles, re-projected to local UTM zone (16N), and converted to 10-m raster. For each resulting raster (Hydro-MEM, GBNERR, NWI), values were reclassified as one of two broad habitat types (Marsh or Not marsh). The Hydro-MEM marsh utilized three productivity levels of low, medium, and high; GBNERR marsh included mid-low marsh, high marsh, and salt panne habitat; and NWI marsh was defined to include estuarine intertidal emergent persistent vegetation, unconsolidated shore, and tidal-fresh palustrine emergent persistent vegetation habitats.

Each resulting land cover (LC) raster was subtracted from the reclassified Hydro-MEM (HM) raster. The resulting difference rasters had values corresponding to areas of agreement (HM Marsh, LC Marsh) and disagreement (HM Not marsh, LC Marsh; HM Marsh, LC Not marsh). The area of each type of agreement or disagreement was calculated and expressed as a percentage of the total marsh area covered by the two rasters being compared (HM and LC). In addition, agreement was quantified by calculating Cohen’s Kappa statistic *K* [91–93]. Validations were restricted to GBNERR boundaries in order to provide a common basis for comparison.

### Marsh migration

Hydro-MEM generates salt marsh biomass density in regularly flooded lands, defined as elevations between MLW and MHW [25,30]. Using the time-stepping framework and feedback mechanism in the Hydro-MEM model, the potential regions for marsh productivity and migration were captured under different SLR scenarios. However, these potential regions included developed areas, agricultural and hay lands, and forests. Marsh productivity projections were used and compared with National Land Cover Data 2011 [94] to produce marsh migration potential maps. The National Land Cover Data 2011 consists of different classifications including water, wetlands, developed area, haylands, and forests that was employed for comparison with the Hydro-MEM projected spatial maps. The comparison detected the areas that salt marsh can migrate to (e.g. forests and agricultural or haylands) or was halted from migration because of hardened structures (e.g. developed areas or highways) as well as inundated regions. This method can detect the potential upland migration paths for marsh systems and other regularly inundated regions under SLR scenarios.

### Storm surge simulations

Storm surge simulations were performed for the Grand Bay estuary in order to determine the relative changes in peak water levels and surge attenuation under each SLR scenario using static and dynamic modeling approaches. The static landscape model assumed no change to the landscape (i.e. bottom friction, topography, or bathymetry) under SLR. The dynamic landscape model incorporated simulated marsh accretion and biomass productivity from the Hydro-MEM to adjust topographic elevations (topography and bathymetry) and bottom friction for the year 2100. In particular, the elevation change in marsh platform and creation of interior ponds from the model outputs were applied under the low and intermediate-low SLR scenarios (Fig 2c and 2d). Bathymetry in the bays were also altered to depict the expansion of the bay under the higher SLR scenarios as obtained in Hydro-MEM results, particularly in the increased expanse of area inundated at MHW (Fig 2e and 2f). Bottom friction was adjusted for wetland regions in a similar fashion as described for the Hydro-MEM simulations and was based on simulated biomass productivity. Using the static and dynamic landscape models for the year 2100, two storm surge simulations for each SLR scenario were performed, each forced by a synthetic storm that directed relatively high water levels to the Grand Bay region (Fig 1a). Storm 1 had a minimum central pressure of 920.30 mb, radius to maximum winds of 14.7 km, and a forward speed of 5.94 m/s and Storm 2, had a minimum central pressure of 938.07 mb, radius to maximum winds of 32.45 km, and a forward speed of 9.67 m/s.

A storm surge transect analysis was performed along a selected location of the Grand Bay marsh (Fig 2). The 10 km transect began at the shoreline and extended upland across the wetland and into the floodplain. Simulated peak water levels and Manning’s *n* values were linearly extracted from the model results at 95 locations spaced 100 m apart. Storm surge attenuation was computed across the transect and defined as the maximum reduction in peak water level for a given distance across the transect [3]. It was quantified by calculating the difference in peak water level from the starting point to each location along the transect and then divided by each point respective distance to the starting point. This resulted in the maximum surge attenuation at each location across the transect relative to the computed surge at the shoreline.

## Results

The evolution of two unique microtidal marsh systems in response to SLR due to the hydrodynamic differences were investigated. The hydrodynamic results for current condition showed a relatively constant value for MHW within the marine-dominated estuary (Fig 3a) in Grand Bay with exception of several tidal creeks. However, MHW in the mixed estuarine system in Weeks Bay (Fig 3b) demonstrated a 2 cm difference (5 percent of the tide range) between Bon Secour Bay and Weeks Bay due to the effect of the narrow inlet between the two bays. Salt marsh productivity results depicted a large marsh area (36 km^2^) in Grand Bay and small patches of marsh lands in Weeks Bay (1.8 km^2^) categorized as low (2 km^2^ in Grand Bay and 0.3 km^2^ in Weeks Bay), medium (13 km^2^ in Grand Bay and 0.7 km^2^ in Weeks Bay), and high (21 km^2^ in Grand Bay and 0.8 km^2^in Weeks Bay) productivity (Fig 3c and 3d).

**Fig 4:**
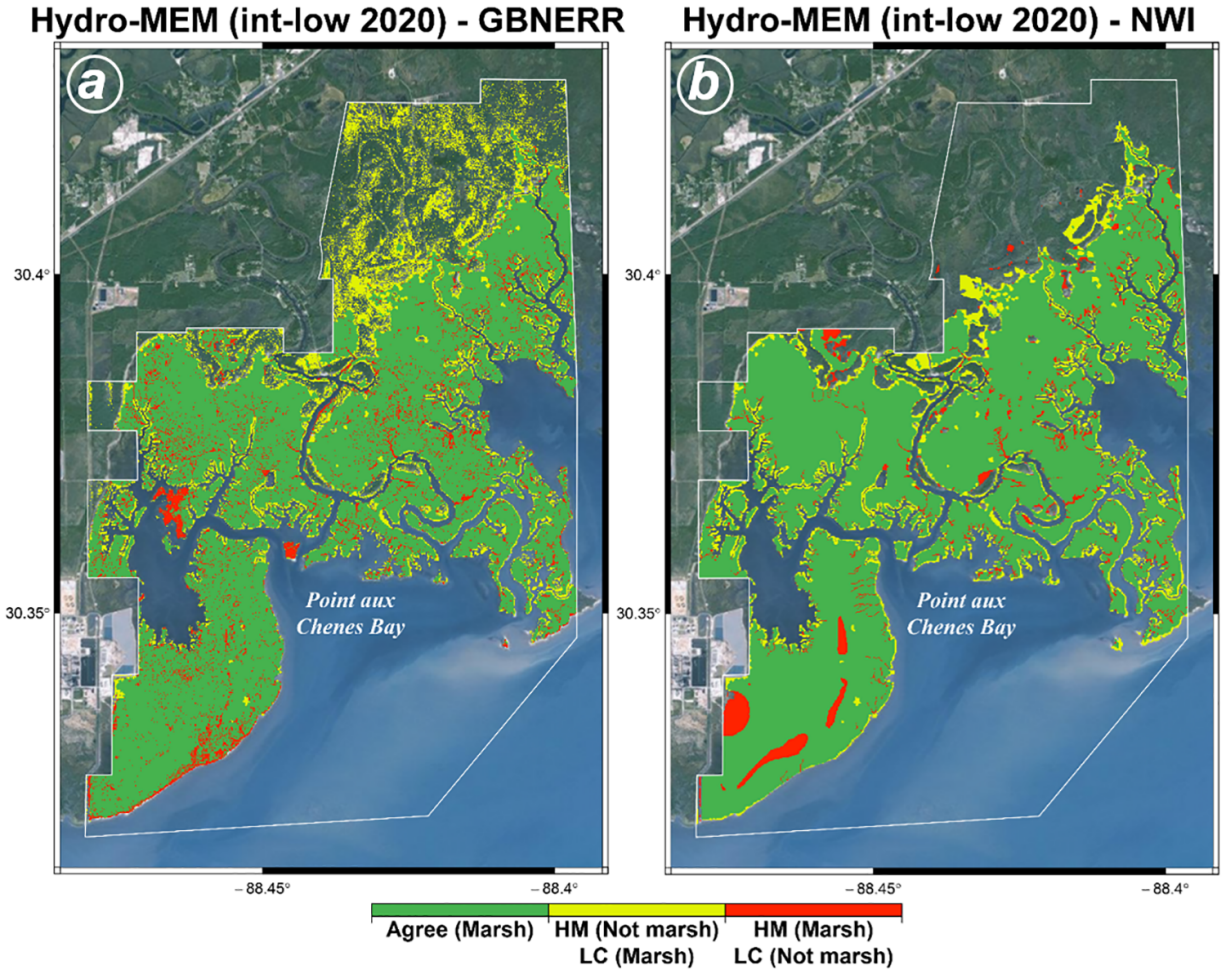
Comparison of the Hydro-MEM results for the year 2020 under the int-low SLR scenario in Grand Bay with high-resolution land cover source of Grand Bay National Estuarine Research Reserve [88] (a) and National Wetlands Inventory [89] (b). The comparison maps are classified as Green (where the model results agree with the land cover map), yellow (where the model does not show marsh but the land cover map shows marsh), and red (where the model shows marsh and land cover map does not show marsh). The basemaps in this figure are screen captures of World Imagery map in ArcGIS [64].

Comparisons between Hydro-MEM results under the intermediate-low 2020 (5 cm) SLR scenario, which were consistent with the lower 95% confidence limit of the observed mean sea level trend at Dauphin Island, and land cover [88,89], as well as the corresponding areas of agreement or disagreement, are illustrated in Fig 4. When compared against GBNERR habitat classifications, 75% of the total combined marsh area was represented by habitat classified as marsh by both sources. Seventeen percent was classified as marsh by GBNERR but not by Hydro-MEM (yellow), and 8% was classified as marsh by Hydro-MEM but not by GBNERR (Fig 4a). In comparing Hydro-MEM to NWI, 82% of the combined marsh area was in agreement, while 13% was classified as marsh by NWI but not by Hydro-MEM, and 5% was classified as marsh by Hydro-MEM but not by NWI (Fig 4b). Cohen’s Kappa statistic [91–93] was calculated as *K* = 0.75 and 0.83 in comparing Hydro-MEM to GBNERR and NWI, respectively. It has been suggested that values of kappa (*K*) greater than about 0.60 indicate ‘substantial’ agreement [92], while values in the range 0.70–0.85 are considered ‘very good’ [93]. In relation to the GBNERR habitat data, areas of disagreement corresponded mainly to marsh areas associated with the transition from estuarine to tidal and freshwater marshes (HM Not marsh, LC Marsh: 17%) or to patchy, shrub-scrub habitat (HM Marsh, LC Not marsh: 8%) (Fig 4a). While the GBNERR habitat classifications do not distinguish between estuarine, tidal, and freshwater marsh habitats, the NWI classifications do make such a distinction. In the comparison between Hydro-MEM and NWI, inclusion of only estuarine and tidal marshes resulted in a larger area of agreement (and smaller areas of disagreement). As with the GBNERR comparison, most areas of disagreement were along the tidal fresh transition zone (HM Not marsh, LC Marsh: 13%) or patchy shrub-scrub areas (HM Marsh, LC Not marsh: 5%), although some of the former disagreement types also appears to occur along areas of transition between marsh and deep water. This may be due, at least in part, to the temporal delay between the imagery on which the NWI classifications are based (2006–2007) and the intermediate-low 2020 Hydro-MEM projections, given that the observed amount of SLR at Dauphin Island between 2007 and 2020 is approximately 4.5 cm (3.7–5.4 cm, based on 3.5 mm/yr +/- 0.62 mm/yr; [90]).

**Fig 5:**
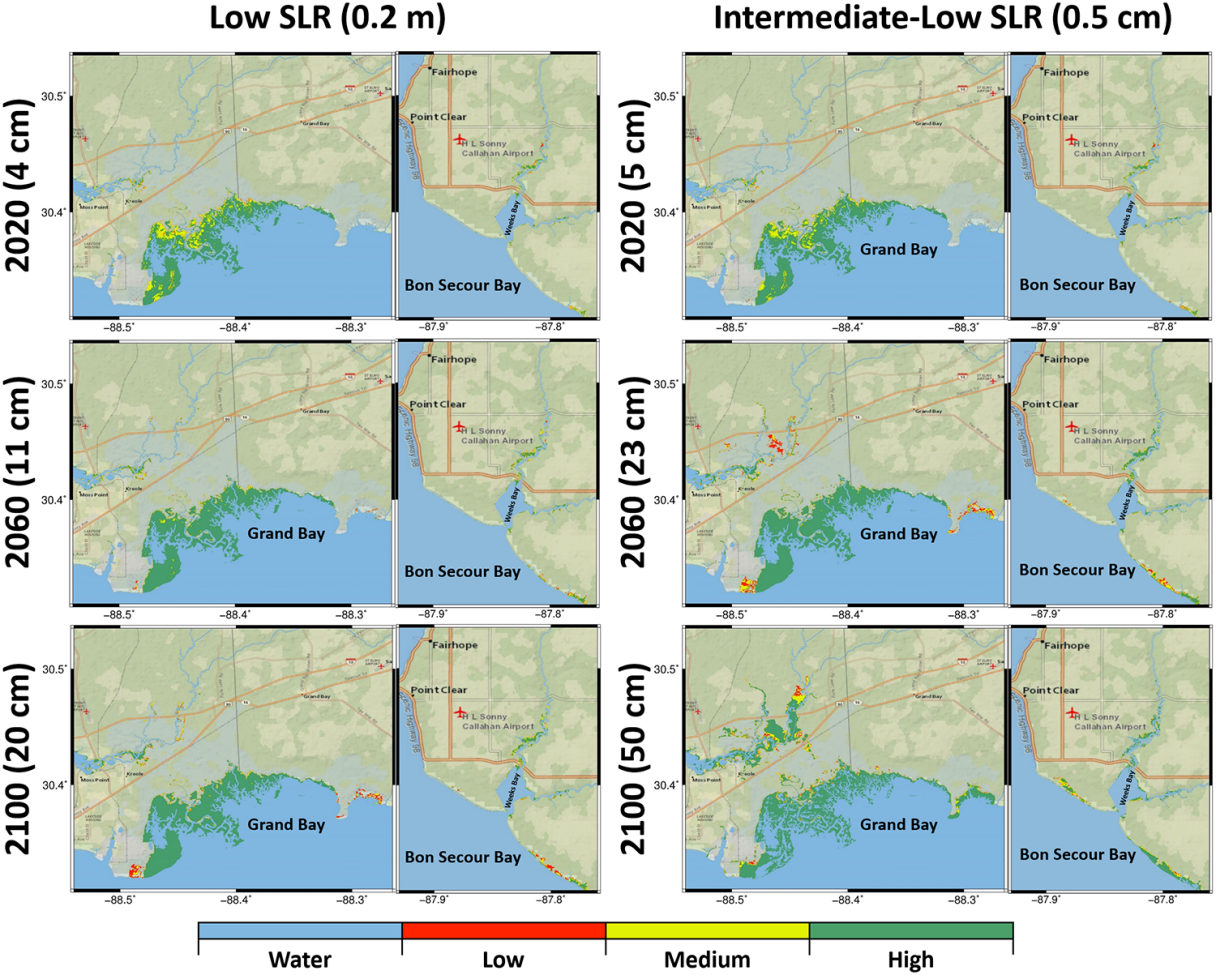
Biomass density results for the low and intermediate-low SLR scenarios categorized into low, medium, and high productivity regions represented by red, yellow, and green, respectively for the Grand Bay and Weeks Bay estuaries. The columns from left to right represent the low and intermediate-low SLR scenarios and each column includes two attached maps demonstrate the Grand Bay and Weeks Bay estuaries, respectively. The rows from top to bottom show the biomass density projections for the year 2040, 2060, and 2100. The basemaps in this figure are screen captures of National Geographic World map in ArcGIS [95].

Fig 5 displays spatial and temporal marsh productivity changes under the low and intermediate-low SLR scenarios for Grand Bay and Weeks Bay estuaries. The changes in marsh productivity under both SLR scenarios followed the same trend in terms of marsh system equilibrium with SLR. In particular, high-productivity marsh coverage in Grand Bay in the year 2100 under the low SLR was demonstrated to increase (double) while no significant change for Weeks Bay was seen. However, the variations over time under the intermediate-low SLR were more noticeable than the low SLR scenario for both Weeks Bay and Grand Bay. The higher marsh productivity in Grand Bay and Weeks Bay (shown as green in the map, Fig 5) was more pronounced between 2060 and 2100 compared to previous years under the intermediate-low SLR scenarios when sea level is projected to increase from 20 to 50 cm. However, in the year 2100 under the intermediate-low SLR scenario in GB, the creation of ponds in the marsh system as well as the bay expansion over the marsh in some regions indicated the start of marsh system loss in this year. The increase in high-productivity marsh land under the intermediate-low scenario in Weeks Bay was more pronounced (approximately a seven-fold increase in coverage in the year 2100) by the creation of new marsh area in Weeks Bay near Bon Secour Bay from 2020 to 2100.

**Fig 6:**
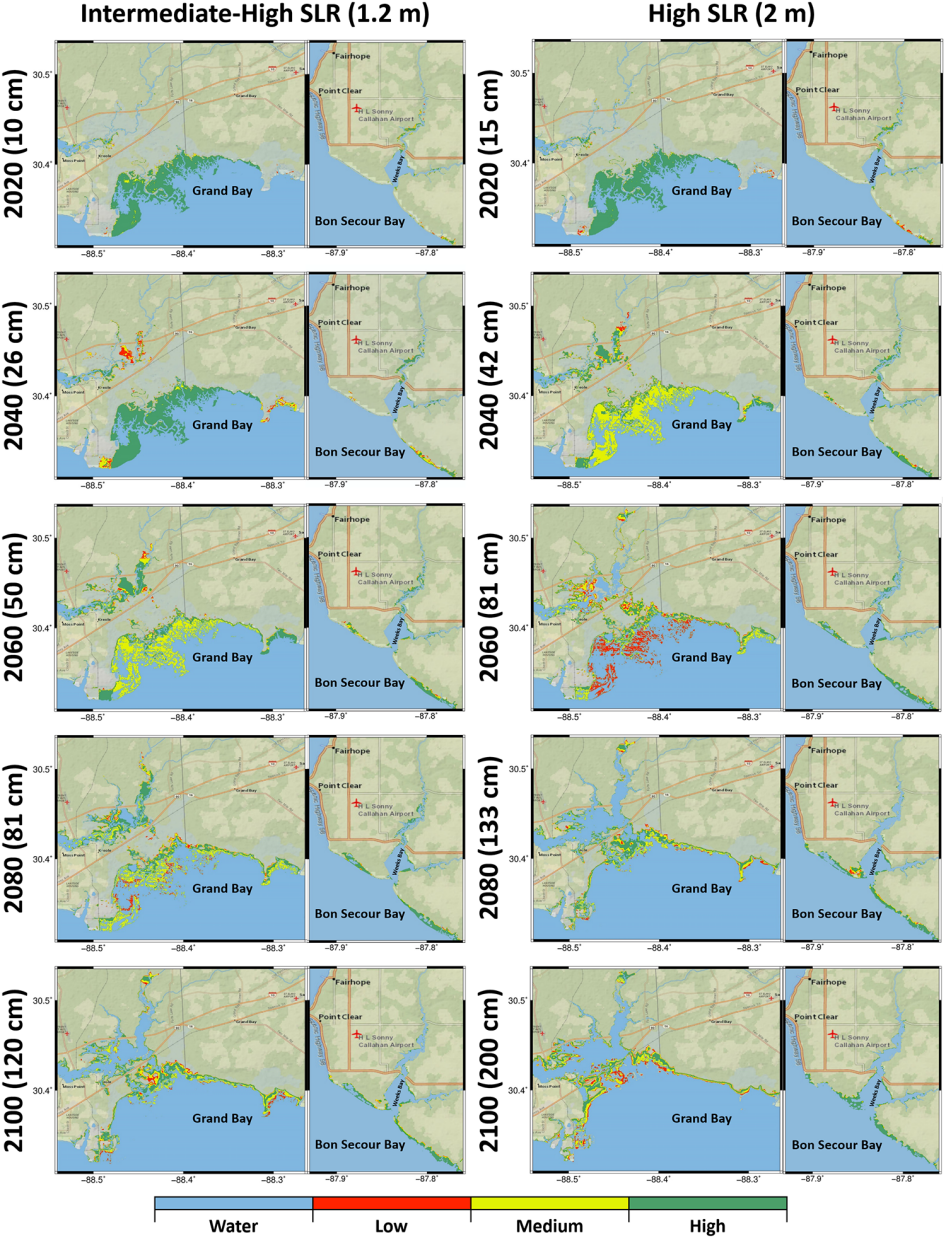
Biomass density results categorized into low, medium, and high productivity regions represented by red, yellow, and green, respectively for the Grand Bay and Weeks Bay estuaries. The columns from left to right represent the intermediate-high and high SLR scenarios and each column includes two attached maps demonstrate the Grand Bay and Weeks Bay estuaries, respectively. The rows from top to bottom show the biomass density projections for the year 2020, 2040, 2060, 2080 and 2100. The basemaps in this figure are screen captures of National Geographic World map in ArcGIS [95].

**Fig 7:**
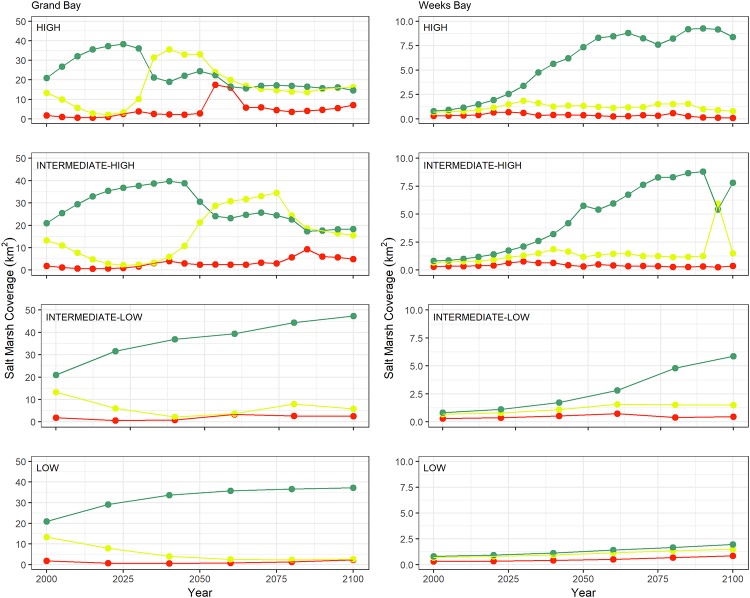
Marsh productivity coverage change graphs. The graphs categorized in low, medium, and high represented by red, yellow, and green lines from 2000 to 2100 for the Grand Bay (left column) and Weeks Bay (right column) estuaries under the low, intermediate-low, intermediate-high, and high SLR scenarios listed from bottom to top row, respectively.

The intermediate-high and high SLR scenarios (1.2m and 2m) projections (Fig 6) showed more dramatic spatial changes in time than the low and intermediate-low SLR scenarios (0.2m and 0.5m) (Fig 5). In the year 2040 projection, under the intermediate-high SLR scenario, the Grand Bay marsh system contained approximately 40 km^2^ of high-productivity marsh compared to 19 km^2^ under the high SLR scenario (Figs 6 and 7). However, 35 km^2^ of medium productivity was computed under the high SLR scenario and indicated the start of marsh productivity loss in the Grand Bay marsh system. Under both intermediate-high and high SLR scenarios, the trend of losing marsh productivity in the Grand Bay estuary was predicted to continue in the year 2060. In this year, high-productivity marsh coverage dropped to 23 km^2^ and medium-productivity marsh coverage jumped to 31 km^2^ under the intermediate-high SLR scenario. Under the high SLR scenario, the medium and high-productivity marsh coverage dropped to 20 km^2^ and low-productivity marsh coverage increased to 16 km^2^ (Figs 6 and 7). The coastline around the Grand Bay estuary also changed by 2100 under these two SLR scenarios, with much of the southern-most salt marsh in the years 2080 to 2090 converting to open water. In addition, Grand Bay became hydraulically connected to the Escatawpa River over Highway 90 in the high SLR scenario. In contrast, the maps in Fig 6 show that the Weeks Bay inlet is projected to become wider to allow more inundation; as a result, marsh regions around the rivers lose productivity and new marsh between Bon Secour Bay and Weeks Bay is created.

For both estuaries, the total salt marsh coverage in 2100 of all four SLR scenarios was estimated to exceed the initial salt marsh coverage (c. 2000, Grand Bay: 36, Weeks Bay: 1.8 km^2^). In 2100, Grand Bay salt marsh coverage totals ranged from 38 km^2^ (high SLR scenario) to 56 km^2^ (intermediate-low SLR scenario). In Weeks Bay, the total salt marsh coverage in 2100 ranged from 10 km^2^ (intermediate-high SLR scenario) to 4 km^2^ (low SLR scenario). While the idealized total salt marsh coverage increased from c. 2000 to 2100, the proportional zonation of salt marsh biomass productivity categories (low, medium, and high) was projected to change dramatically over time and between the two evaluated estuaries (Fig 7).

Grand Bay transitioned from an environment composed of high and medium productivity in the initial time (c. 2000) to one dominated in 2100 by high productivity (low and intermediate-low SLR scenarios) or to conditions similar to the starting environment with additional low productivity marsh coverage (high and intermediate-high SLR scenarios, Fig 7). In the high and intermediate-high SLR scenarios between 2000 and 2100, there were periods of steep coverage increase and decline for each productivity category as new areas transitioned to marsh with new inundation while lower-lying areas fragmented and converted from salt marsh to open water.

Weeks Bay transitioned from similar coverages of medium (0.7 km^2^) and high (0.8 km^2^) marsh productivity in c. 2000, to an estuary dominated by high marsh productivity (intermediate-low, intermediate-high, and high SLR scenarios, Fig 7). In the low SLR scenario, the ratio of low (20%), medium (35%), and high (45%) productivity in 2100 remained similar to that of 2000 (low: 17%, medium: 38%, high: 45%); however, there was an increase in coverage for all three categories over time. Moreover, the sharp change in the year 2095 in the Weeks Bay estuary under the intermediate-high SLR scenario (Fig 7) was projected to occur due to the loss of productivity from high to medium near the shorelines in the southern part of Bon Secour Bay where the new marsh land was created. This medium productivity region in the year 2100 was estimated to become inundated as a result of high SLR and some migration to higher lands was predicted (Fig 6).

**Fig 8:**
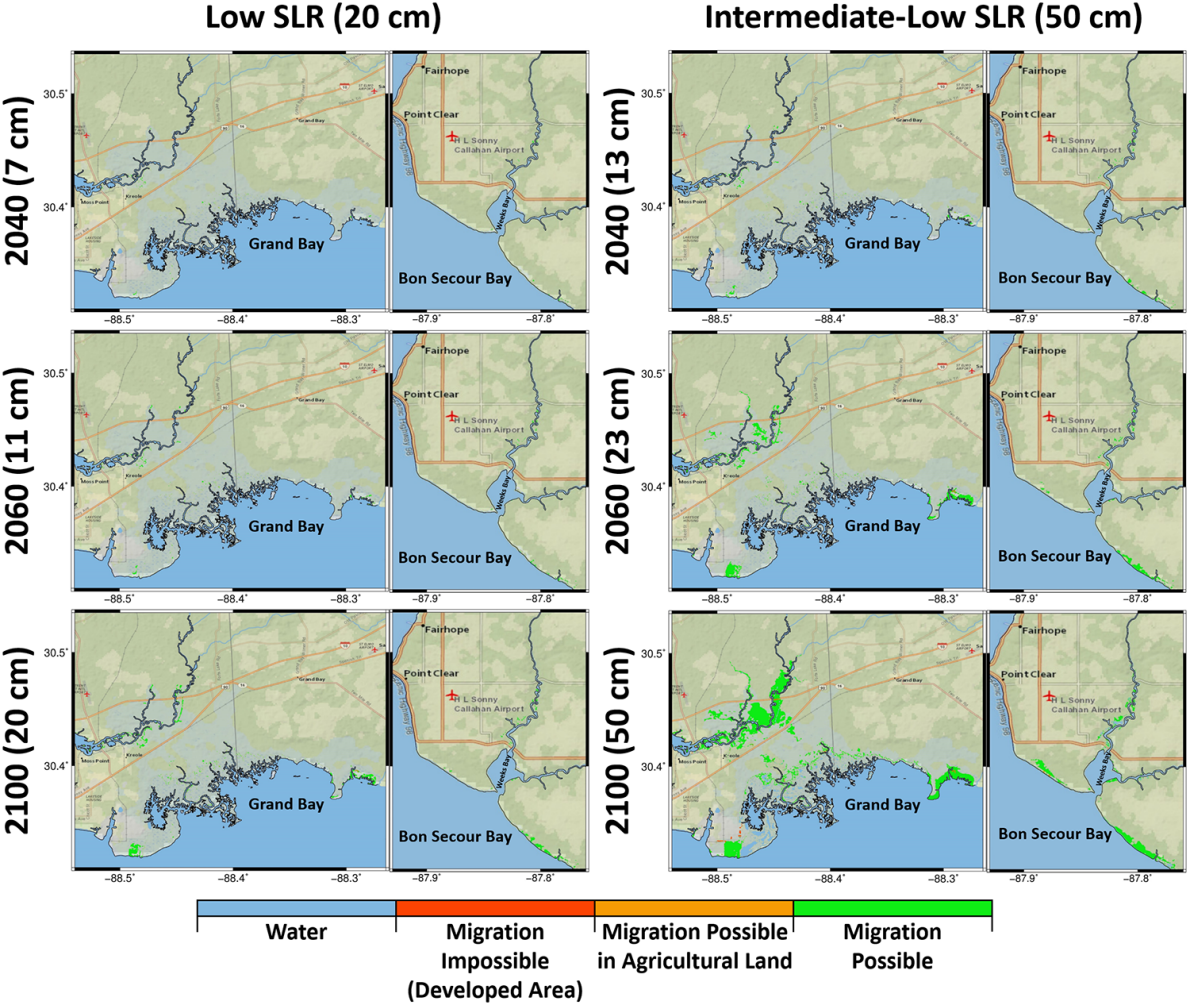
Marsh migration potential maps for the low and intermediate-low SLR scenarios categorized into the water, migration-impossible, migration-possible in agricultural land, and migration-possible regions represented by blue, red, orange, and green, respectively for the Grand Bay and Weeks Bay estuaries. The columns from left to right represent the low and intermediate-low SLR scenarios and each column includes two attached maps demonstrate the Grand Bay and Weeks Bay estuaries, respectively. The rows from top to bottom show the biomass density projections for the year 2040, 2060, and 2100. The basemaps in this figure are screen captures of National Geographic World map in ArcGIS [95].

**Fig 9:**
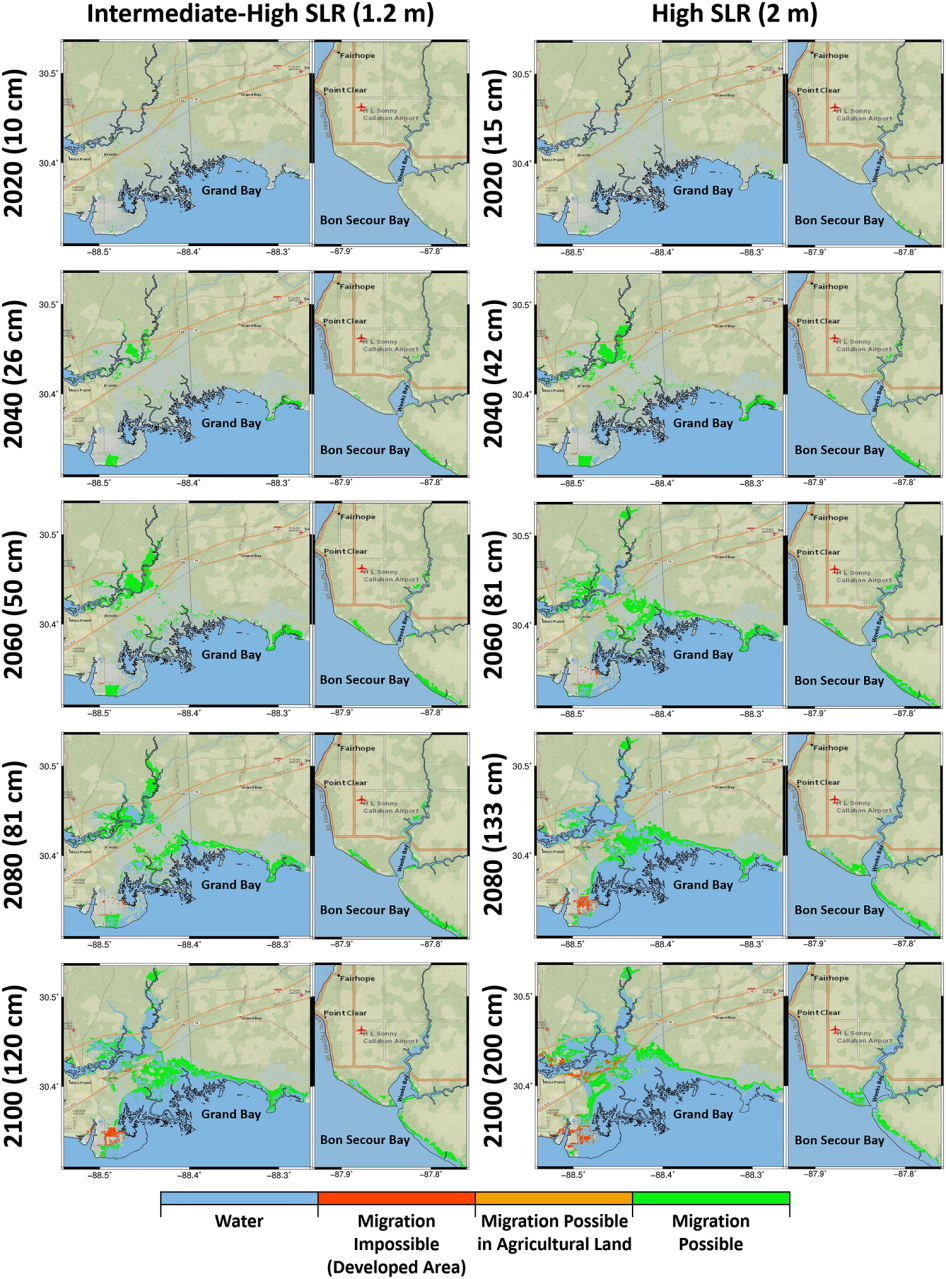
Marsh migration potential maps for the intermediate-high and high SLR scenarios categorized into the water, migration-impossible, migration-possible in agricultural land, and migration-possible regions represented by blue, red, orange, and green, respectively for the Grand Bay and Weeks Bay estuaries. The columns from left to right represent the intermediate-high and high SLR scenarios and each column includes two attached maps demonstrate the Grand Bay and Weeks Bay estuaries, respectively. The rows from top to bottom show the biomass density projections for the year 2020, 2040, 2060, 2080 and 2100. The basemaps in this figure are screen captures of National Geographic World map in ArcGIS [95].

Figs 8 and 9 present the potential for marsh migration in Grand Bay and Weeks Bay. Under the low SLR scenario in both estuaries, the increase in the green area (the region where marsh migration is possible) was much smaller compared to other scenarios. Under the intermediate-low SLR scenario, not only do the potentially suitable regions for marsh migration expanded in time but the map also includes small patches of marsh migration potential into hay lands and developed areas (depicted by orange and red colors representing impossibility of marsh migration to those regions). These migration-impossible regions were highlighted as marsh migration obstruction under the intermediate-high and high SLR scenarios from the year 2060 to 2100 (Fig 9). The maps also show the flooded regions in blue under the intermediate-high and high SLR scenarios between the current shoreline border and migrated marsh system.

**Fig 10:**
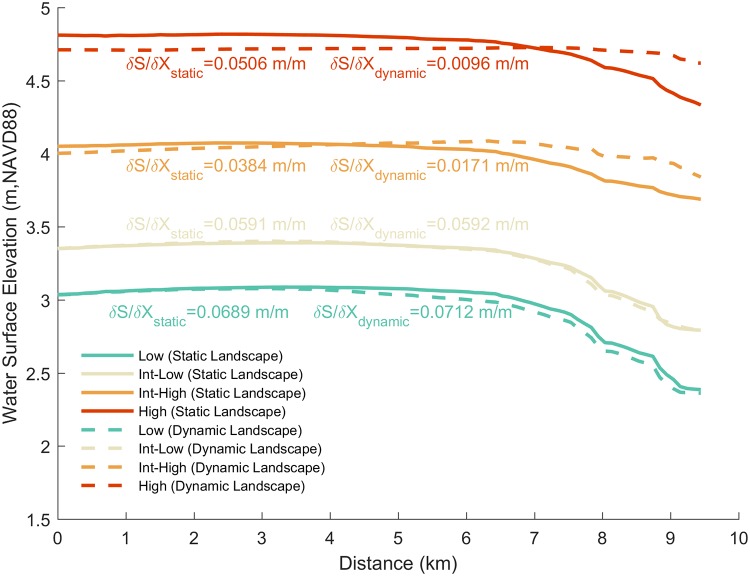
Simulated peak storm surge water surface elevation profile across the Grand Bay marsh (Fig 2 for transect location). Transect distance start from the present day shoreline (0 km) and extent north into the marsh up to 9.5 km. The maximum surge attenuation, *δS* / *δX*, are shown for each SLR scenario and for the static and dynamic landscape.

Fig 10 shows the maximum water surface elevation profile across the Grand Bay estuary under each SLR scenario, including the results from the static and dynamic landscape, for Storm 1. As SLR increased, the average total water level across the transect also increased, but in a non-linear fashion, meaning the total water level did not linearly increase by the amount of SLR applied, for both the static and dynamic landscape experiments. For the dynamic case, the increase in total water level at the start of the transect (0 km) was 0.32 m from low to intermediate-low (linear offset value of 0.3 m), 0.65 m from intermediate-low to intermediate-high (linear offset value of 0.7 m), and 0.71 m from intermediate-high to high (linear offset value of 0.8 m), which represented 9.5%, 16.3%, and 15.0% of the total water level, respectively. The attenuation across the transect for the static and dynamic landscapes under the low and intermediate-low scenarios were similar, although slight variations were found in the water surface elevation profile in the upland regions (5–10 km). More prominent changes between the static and dynamic landscape representations were observed in the water surface elevation profile for the intermediate-high and high scenarios, particularly the water level in the upland portions of the transect (5–10 km). Additionally, surge attenuation was reduced in the static to dynamic landscape change scenario under the intermediate-high and high scenarios from 0.038 m/m to 0.017 m/m (55.5% reduction) and 0.050 m/m to 0.009 m/m (81.0% reduction), respectively.

**Fig 11:**
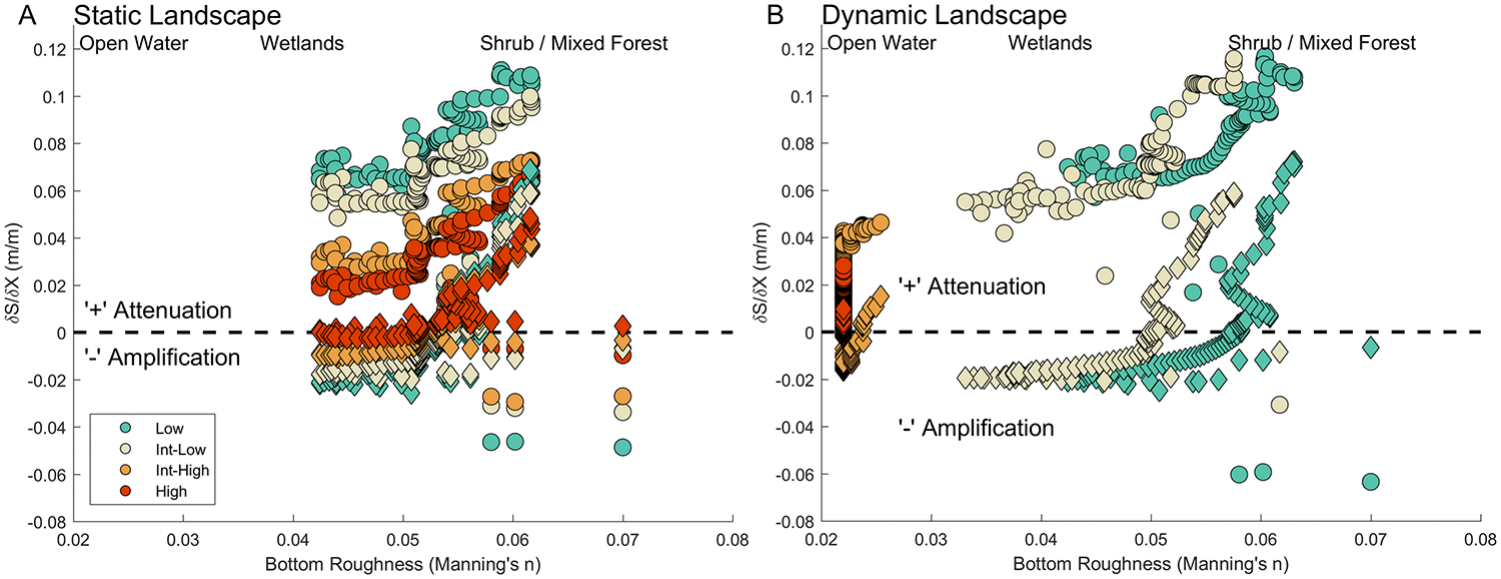
Storm surge attenuation as a function of bottom roughness (via Manning’s n) in Grand Bay for two hurricane events (circles and diamonds) using A) static landscape and B) dynamic landscape (altered bottom roughness and topography/bathymetry). Negative values indicate storm surge amplification (water level values increasing along the transect–see Fig 2 for location) and positive values indicate storm surge attenuation (water levels reducing along the transect).

Fig 11 displays the storm surge attenuation as a function of Manning’s *n* bottom roughness coefficient for Storm 1 and 2 and for the static and dynamic landscape experiments under the four SLR scenarios. The maximum surge attenuation increased with increasing bottom roughness as one would expect. For the static case, an increase in sea level reduced the surge attenuation, particularly as the roughness increased beyond a Manning’s *n* value of 0.055; results from the lower SLR scenarios demonstrated larger values of attenuation across the transect. However, this pattern changed under the dynamic landscape experiment and the intermediate-low provided increased attenuation compared to the low scenario for similar bottom roughness.

## Discussion

Estuaries are tightly connected to relative SLR; they can expand under low rates of SLR or become submerged under high rates of SLR [96]. Microtidal estuaries around the world and their wetlands are more vulnerable to SLR than macrotidal estuaries; they tend to have large areas of low-land marsh which are susceptible to drowning with increased SLR flooding [7–9,97]. The vulnerability of estuaries to SLR varies based on the topographical and hydrodynamic characteristics, as well as sediment inputs. To understand the effects of these underlying drivers on the response to SLR, it is helpful to compare and contrast the main types of microtidal estuaries, namely fluvial, marine, and mixed. This study investigated how major characteristics of a marine and mixed microtidal estuary influence their response to SLR, compared them to each other and to a fluvial system.

The main hydrodynamic difference between marine-dominated and mixed estuaries is the fluvial source. Marine-dominated estuaries lack a fluvial source and the associated interaction between river and tidal flows. However, mixed estuaries receive fluvial effects including the sediments and water level changes within the creek networks. These effects are much less significant than those in fluvial estuaries where river flows are relatively large and the creek networks are more complex and often contains distributaries from the main river [15,28]. Estuaries that are influenced by river input generally demonstrate greater water level variation between the creeks and the bay. The tide and river inflow interaction creates a more complex estuarine geometry, as evidenced by Weeks Bay (Fig 3b) [28]. The lack of river inflow in marine-dominated estuaries results in simple creek networks with a relatively uniform water level between the creeks and the bay; Grand Bay is an example of this type of system (Fig 3a). There, the MHW change between the creeks and the bay in Grand Bay was minor, whereas the MHW variation between Bon Secour Bay and Weeks Bay illustrates how the narrow inlet between the two bays dampens the tidal signal, currents, waves, and storm surge. Research shows that there is a correlation between SLR, the change in inlet cross-section area, tidal amplitude, and tidal asymmetry [83,98]. The complex geometry of an estuary can dampen the effects of SLR in these mixed estuaries, as long as the river discharge does not contribute disproportionately to the inundation of the marsh. However, in a marine-dominated estuary, the simple creeks and uniform water level are more directly influenced by tidal flow, which increases with SLR. In contrast, the river inflow in a fluvial estuary can exacerbate marsh inundation when combined with increased tidal flow as a result of SLR due to the increase in downstream water level and the associated reduction in the hydraulic gradient [28].

In marine-dominated estuaries, spatially constant MHW elevations in the tidal creeks and flat topographic gradients combine, create, and sustain extensive marsh land (Fig 3). Paradoxically, these same characteristics make them vulnerable to SLR (Fig 6). In mixed estuaries, the steeper topographic variations, including barriers, can limit marsh expansion potential. As an example, small patches of marsh regions in the Weeks Bay mixed estuary (Fig 3d) demonstrate how these steeper topographical variations limit the exposure of low-land regions to tidal flow and cause pockets of marsh migration rather than large swaths. The tidal flows in Weeks Bay are also damped by the narrow inlet, an important topographical feature. The increased exposure that comes along with the wider inlet cross section induced by SLR [83] can introduce larger tide range and consequently broaden intertidal lands to create new marsh regions in the future (Figs 5 and 6).

The response of different types of estuaries to SLR depends on both SLR rate and the characteristics of the estuaries. In marine-dominated estuaries, the low and uniform land in addition to smooth increase in elevation from low to high marsh provide a somewhat optimal situation for the marsh system to establish an equilibrium under the lower SLR scenarios. This equilibrium between the marsh system and SLR in the marine-dominated estuaries can expand marsh lands and increase marsh productivity. This equilibrium was shown in our study in the marine-dominated estuary under both low and intermediate-low-SLR scenarios (Figs 5 and 7). Examining productivity and marsh migration maps (Figs 5 and 8), the results demonstrate simultaneous marsh migration and ponding under intermediate-low SLR scenario. Both ponding and marsh migration can be managed by restoration activities such as thin layer placement and land preparation. In mixed estuaries, regular cyclical flooding of higher lands is more likely to occur under the intermediate-low SLR scenario, which can result in marsh system expansion into new territory that lies in the optimal vertical position of the tidal frame. In this study, the selected mixed estuary projections for the intermediate-low SLR scenario (Figs 5 and 8) showed marsh migration near Fish River in addition to the creation of new productive marsh areas (increase in high productivity marsh in Fig 7) near Bon Secour Bay where water levels increased. In addition, the combination of river inflow and SLR under the intermediate-low SLR in both mixed and fluvial microtidal estuaries can cause ponding and marsh migration [28].

The microtidal estuaries’ response to higher rates of SLR is more dramatic. Marine-dominated estuaries are the most vulnerable to higher SLR rates because of their low uniform topography and low accretion rates (due to the lack of sediment source). This research showed that marshes in this type of estuaries lose their productivity faster than those in mixed estuaries but slower than those in fluvial estuaries [28], where the loss in productivity is due to increased inundation of the marsh system induced by the synergistic effects of SLR and river discharge. For example, the high productivity areas in Grand Bay were projected to become medium productivity under intermediate-high (the year 2055 in Fig 7 and the year 2060 in Fig 6) and high (from the year 2035 in Fig 7 and the year 2040 in Fig 6) SLR scenarios. This finding is similar, for example, to estuaries in the Mediterranean Sea where the marine-dominated marsh sites were shown to be more vulnerable to SLR greater than 1 m. Marshes in marine-dominated estuaries in other parts of the world also lose productivity as a result of increased inundation of marsh platforms, lower accretion rates, and lack of sediment inputs compared to the other types of estuaries [9]. Moreover, the migration and coverage results (Figs 5, 7 and 9) show that on a gently sloping coastal plain, the rate of migration in response to SLR can be substantial in the absence of natural or anthropogenic topographic barriers, which agrees with other similar studies [47,49,50]. Characteristics of mixed estuaries, such as a gradual slope to higher lands, hydrodynamics dampened by the complex geometry, and sediment transported by small rivers, lower the rate of marsh system inundation. In mixed estuaries, the sediment availability and the suitable land slope provide the potential for marsh accretion, upland marsh migration, and creation of new wetlands.

This research presented an idealized case where salt marsh is able to migrate to higher lands. However, some of these regions are bordered by developed area or agricultural / hay lands and would require restoration to facilitate the migration. Marsh migration-potential maps can help highlight the possible regions that can be occupied by salt marsh in an ideal situation. Under the higher SLR scenarios (Fig 9), the vast amount of marsh migration necessary to maintain current coverage would require restoration activities such as buying, protecting, and preparing the land. For example, it is virtually impossible for the marsh to migrate to the higher lands on the west and north side of the current marsh region in Grand Bay because of a hardened surface and a highway (Fig 9). In addition, some smaller patches shaded with orange in the northern part of the Grand Bay estuary and the northern shores of Bon Secour Bay indicate hay lands where marsh could migrate if the lands are saved and prepared for marsh restoration. These maps also highlight the potential future flooded regions by delineating the present day shoreline. These potential future flooded regions could be good candidates for thin layer sediment placement that would allow the marsh platform to better keep pace with SLR. Since Hydro-MEM was designed to simulate large extents of salt marsh and was also applied and validated in two different estuaries in the Timucuan and Apalachicola marsh systems [25,28], it can be used to inform marsh restoration programs in large intertidal wetlands as well as the systems presented here. In particular, the maps of future wetland productivity and migration projections can help coastal resource managers target effective restoration activities in the NGOM and other microtidal systems in the world [7,99].

Changes to the coastal landscape under SLR, such as the expansion of tidally inundated regions (Fig 2) and change in marsh productivity (Fig 6) drastically alter the potential for storm surge attenuation (Figs 10 and 11). The estimated reduction in wetland coverage and bay expansion is projected to cause the total water levels to increase and storm surge to penetrate further inland than under present-day conditions. Reduced storm surge attenuation under future sea levels will increase the vulnerability of coastal communities that are currently protected, whether they realize it or not, by large marsh systems such as Grand Bay. Therefore, it is critical to develop an understanding of how tidal hydrodynamics may change under future SLR and the resulting morphological response of the coastal system [100]. Herein, we implemented simple morphological modifications to determine relative changes, but additional research is needed to better understand the dynamic feedback among the tidal hydrodynamic influenced morphology and resulting ecology [21].

## Conclusions

This study utilized the coupled Hydro-MEM model to examine the impacts of SLR in microtidal marine-dominated (Grand Bay) and mixed (Weeks Bay) estuaries in the northern Gulf of Mexico. The model was initialized with conditions c. 2000 including tidal hydrodynamics and marsh platform topography adjusted to remove lidar DEM bias. The model used the accretion rate to update marsh platform elevation and change bottom friction with respect to marsh productivity variations in time. The model results were validated with the most recent data from the Grand Bay NERR and NWI and showed 82% agreement.

The marsh productivity results in the marine-dominated estuary in Grand Bay show a highly productive marsh system under the low and intermediate-low SLR scenarios with the maximum increase in marsh coverage (from 36 km^2^ in the year 2000 to 56 km^2^ in the year 2100) under the intermediate-low SLR scenario. The results also indicate pond creation in the year 2100 under the intermediate-low SLR scenario, which can be a precursor to losing marsh systems permanently in years following 2100. Although the marsh coverage in the Grand Bay estuary could remain the same under intermediate-high and high SLR scenario if the marsh can migrate to higher lands, the current marsh is projected to become open water by the year 2100.

The unique topography and particular bay characteristics of the mixed estuary in Weeks Bay, as well as sediment sources, benefit the salt marsh system and make it more resilient to SLR. The maximum wetland coverage increase was predicted to occur under the intermediate-high SLR scenario (a five-fold increase from 1.8 km^2^ in the year 2000 to 10 km^2^ in the year 2100). This trend was also projected under the high SLR scenario if the marsh migration to higher lands near the Bon Secure Bay becomes possible. In addition, marsh migration under the intermediate-low SLR scenario in Weeks Bay increased the marsh system coverage area, but the projections show only a slight change under the low SLR scenario.

The Hydro-MEM results were also used to apply changes in bottom friction, marsh platform elevation, and bay expansion in a storm surge model to assess the effects of these variations on storm surge attenuation in Grand Bay. The water level changes indicated an increase in storm surge in the higher lands with less attenuation under the intermediate-high and high SLR scenarios in the year 2100 as a result of the marshes transitioning to open water and ceasing to provide their surge and wave dissipating ecosystem service.

This research presented the effects of SLR on two hydrodynamically-unique estuaries, and its results can help guide future restoration projects in the NGOM and beyond. The Hydro-MEM modeling approach used in this study can be applied to any other marsh system to aid coastal managers. The maps generated and tools used here, as well as lessons learned, can be used to inform decisions with respect to SLR impacts on ecological resources, as well as decisions on what to look for and where to restore developed land and provide potential migration sites.

## References

[1] Yoskowitz D, Carollo C, Pollack JB, Santos C, Welder K. Integrated ecosystem services assessment: Valuation of changes due to sea level rise in Galveston Bay, Texas, USA. Integrated Environmental Assessment and Management. 2017;13(2):431–43. 10.1002/ieam.1798 27249782

[2] Barbier EB, Hacker SD, Kennedy C, Koch EW, Stier AC, Silliman BR. The value of estuarine and coastal ecosystem services. Ecological Monographs. 2011;81(2):169–93. 10.1890/10-1510.1

[3] Barbier EB, Georgiou IY, Enchelmeyer B, Reed DJ. The Value of Wetlands in Protecting Southeast Louisiana from Hurricane Storm Surges. PLOS ONE. 2013;8(3):e58715 10.1371/journal.pone.0058715 23536815 PMC3594144

[4] Turner RE. Wetland loss in the Northern Gulf of Mexico: Multiple working hypotheses. Estuaries. 1997;20(1):1–13. 10.2307/1352716

[5] Thieler ER, Hammer-Klose ES. National Assessment of Coastal Vulnerability to Sea Level rise: Preliminary Results for the U.S. Atlantic Coast. Woods Hole, Massachusetts: US Geological Survey, 1999.

[6] Nicholls RJ, Hoozemans FMJ, Marchand M. Increasing flood risk and wetland losses due to global sea-level rise: regional and global analyses. Global Environmental Change. 1999;9, Supplement 1:S69–S87. 10.1016/S0959-3780(99)00019-9.

[7] Osland MJ, Griffith KT, Larriviere JC, Feher LC, Cahoon DR, Enwright NM, et al Assessing coastal wetland vulnerability to sea-level rise along the northern Gulf of Mexico coast: Gaps and opportunities for developing a coordinated regional sampling network. PLOS ONE. 2017;12(9):e0183431 10.1371/journal.pone.0183431 28902904 PMC5597133

[8] Day J, Pont D, Hensel P, Ibañez C. Impacts of sea-level rise on deltas in the Gulf of Mexico and the Mediterranean: The importance of pulsing events to sustainability. Estuaries. 1995;18(4):636–47. 10.2307/1352382

[9] Day J, Ibáñez C, Scarton F, Pont D, Hensel P, Day J, et al Sustainability of Mediterranean Deltaic and Lagoon Wetlands with Sea-Level Rise: The Importance of River Input. Estuaries and Coasts. 2011;34(3):483–93. 10.1007/s12237-011-9390-x

[10] Friedrichs C, Aubrey D, Speer P. Impacts of Relative Sea-level Rise on Evolution of Shallow Estuaries In: Cheng RT, editor. Residual Currents and Long-term Transport. Coastal and Estuarine Studies. 38: Springer New York; 1990 p. 105–22.

[11] Townend I, Pethick J. Estuarine flooding and managed retreat. Philosophical Transactions of the Royal Society of London A: Mathematical, Physical and Engineering Sciences. 2002;360(1796):1477–95. 10.1098/rsta.2002.1011 12804261

[12] Rilo A, Freire P, Guerreiro M, Fortunato AB, Taborda R. Estuarine margins vulnerability to floods for different sea level rise and human occupation scenarios. Journal of Coastal Research. 2013;Special Issue No. 65:820–5.

[13] Kästner K, Hoitink AJF, Vermeulen B, Geertsema TJ, Ningsih NS. Distributary channels in the fluvial to tidal transition zone. Journal of Geophysical Research: Earth Surface. 2017;122(3):696–710. 10.1002/2016JF004075

[14] Savenije HH. Salinity and tides in alluvial estuaries: Elsevier; 2006.

[15] Hughes ZJ. Tidal Channels on Tidal Flats and Marshes In: Davis RA Jr, Dalrymple RW, editors. Principles of Tidal Sedimentology. Dordrecht: Springer Netherlands; 2012 p. 269–300.

[16] Broome SW, Seneca ED, Woodhouse WW Jr. Tidal salt marsh restoration. Aquatic Botany. 1988;32(1–2):1–22. 10.1016/0304-3770(88)90085-X.

[17] Wolanski E, Chappell J. The response of tropical Australian estuaries to a sea level rise. Journal of Marine Systems. 1996;7(2–4):267–79. 10.1016/0924-7963(95)00002-X.

[18] Hearn CJ, Atkinson MJ. Effects of sea-level rise on the hydrodynamics of a coral reef lagoon: Kaneohe Bay, Hawaii Sea-level Changes and Their Effects 2001 p. 25–477.

[19] Leorri E, Mulligan R, Mallinson D, Cearreta A. Sea-level rise and local tidal range changes in coastal embayments: An added complexity in developing reliable sea-level index points. Journal of Integrated Coastal Zone Management. 2011;11:307–14.

[20] Passeri D, Hagen S, Bilskie M, Medeiros S. On the significance of incorporating shoreline changes for evaluating coastal hydrodynamics under sea level rise scenarios. Natural Hazards. 2014:1–19. 10.1007/s11069-014-1386-y

[21] Ganju NK, Brush MJ, Rashleigh B, Aretxabaleta AL, del Barrio P, Grear JS, et al Progress and Challenges in Coupled Hydrodynamic-Ecological Estuarine Modeling. Estuaries and Coasts. 2016;39(2):311–32. 10.1007/s12237-015-0011-y 27721675 PMC5053394

[22] D’Alpaos A, Lanzoni S, Marani M, Rinaldo A. Landscape evolution in tidal embayments: Modeling the interplay of erosion, sedimentation, and vegetation dynamics. Journal of Geophysical Research: Earth Surface. 2007;112(F1):F01008 10.1029/2006JF000537

[23] Kirwan ML, Murray AB. A coupled geomorphic and ecological model of tidal marsh evolution. Proceedings of the National Academy of Sciences. 2007;104(15):6118–22. 10.1073/pnas.0700958104 17389384 PMC1851060

[24] Hagen S, Morris J, Bacopoulos P, Weishampel J. Sea-Level Rise Impact on a Salt Marsh System of the Lower St. Johns River. Journal of Waterway, Port, Coastal, and Ocean Engineering. 2013;139(2):118–25. 10.1061/(ASCE)WW.1943-5460.0000177

[25] Alizad K, Hagen SC, Morris JT, Bacopoulos P, Bilskie MV, Weishampel J, et al A coupled, two-dimensional hydrodynamic-marsh model with biological feedback. Ecological Modeling. 2016;327:29–43. 10.1016/j.ecolmodel.2016.01.013.

[26] Schile LM, Callaway JC, Morris JT, Stralberg D, Parker VT, Kelly M. Modeling tidal marsh distribution with sea-level rise: Evaluating the role of vegetation, sediment, and upland habitat in marsh resiliency. PLOS ONE. 2014;9(2):e88760 10.1371/journal.pone.0088760 24551156 PMC3923833

[27] Passeri DL, Hagen SC, Medeiros SC, Bilskie MV, Alizad K, Wang D. The dynamic effects of sea level rise on low gradient coastal landscapes: a review. Earth’s Future. 2015:n/a–n/a. 10.1002/2015EF000298

[28] Alizad K, Hagen SC, Morris JT, Medeiros SC, Bilskie MV, Weishampel JF. Coastal wetland response to sea-level rise in a fluvial estuarine system. Earth’s Future. 2016;4(11):483–97. 10.1002/2016EF000385

[29] Medeiros S, Hagen S, Weishampel J, Angelo J. Adjusting Lidar-Derived Digital Terrain Models in Coastal Marshes Based on Estimated Aboveground Biomass Density. Remote Sensing. 2015;7(4):3507–25. 10.3390/rs70403507

[30] Morris JT, Sundareshwar PV, Nietch CT, Kjerfve B, Cahoon DR. Responses of Coastal Wetlands to Rising Sea Level. Ecology. 2002;83(10):2869–77. 10.1890/0012-9658(2002)083[2869:rocwtr]2.0.co;2

[31] Homer C, Huang C, Yang L, Wylie B, Coan M. Development of a 2001 national land-cover database for the United States. Photogrammetric Engineering & Remote Sensing. 2004;70(7):829–40.

[32] Knutson P. Role of Coastal Marshes in Energy Dissipation and Shore Protection The Ecology and Management of Wetlands: Springer US; 1987 p. 161–75.

[33] Leonard LA, Luther ME. Flow hydrodynamics in tidal marsh canopies. Limnology and Oceanography. 1995;40(8):1474–84. 10.4319/lo.1995.40.8.1474

[34] Möller I, Spencer T. Wave dissipation over macro-tidal saltmarshes: Effects of marsh edge typology and vegetation change. Journal of Coastal Research. 2002;36:506–21.

[35] Shepard CC, Crain CM, Beck MW. The Protective Role of Coastal Marshes: A Systematic Review and Meta-analysis. PLOS ONE. 2011;6(11):e27374 10.1371/journal.pone.0027374 22132099 PMC3223169

[36] Augustin LN, Irish JL, Lynett P. Laboratory and numerical studies of wave damping by emergent and near-emergent wetland vegetation. Coastal Engineering. 2009;56(3):332–40. 10.1016/j.coastaleng.2008.09.004.

[37] Foster-Martinez MR, Lacy JR, Ferner MC, Variano EA. Wave attenuation across a tidal marsh in San Francisco Bay. Coastal Engineering. 2018;136:26–40. 10.1016/j.coastaleng.2018.02.001.

[38] Wamsley TV, Cialone MA, Smith JM, Atkinson JH, Rosati JD. The potential of wetlands in reducing storm surge. Ocean Engineering. 2010;37(1):59–68. 10.1016/j.oceaneng.2009.07.018.

[39] Wamsley TV, Cialone MA, Smith JM, Ebersole BA, Grzegorzewski AS. Influence of landscape restoration and degradation on storm surge and waves in southern Louisiana. Natural Hazards. 2009;51(1):207–24. 10.1007/s11069-009-9378-z

[40] Donnelly JP, Bertness MD. Rapid shoreward encroachment of salt marsh cordgrass in response to accelerated sea-level rise. Proceedings of the National Academy of Sciences. 2001;98(25):14218–23. 10.1073/pnas.251209298 11724926 PMC64662

[41] Rodríguez JF, Saco PM, Sandi S, Saintilan N, Riccardi G. Potential increase in coastal wetland vulnerability to sea-level rise suggested by considering hydrodynamic attenuation effects. Nature Communications. 2017;8:16094 10.1038/ncomms16094 https://www.nature.com/articles/ncomms16094#supplementary-information. 28703130 PMC5511368

[42] Mariotti G. Revisiting salt marsh resilience to sea level rise: Are ponds responsible for permanent land loss? Journal of Geophysical Research: Earth Surface. 2016;121(7):1391–407. 10.1002/2016JF003900

[43] Schepers L, Kirwan M, Guntenspergen G, Temmerman S. Spatio-temporal development of vegetation die-off in a submerging coastal marsh. Limnology and Oceanography. 2017;62(1):137–50. 10.1002/lno.10381

[44] Spalding EA, Hester MW. Interactive effects of hydrology and salinity on oligohaline plant species productivity: Implications of relative sea-level rise. Estuaries and Coasts. 2007;30(2):214–25. 10.1007/BF02700165

[45] Mariotti G, Canestrelli A. Long-term morphodynamics of muddy backbarrier basins: Fill in or empty out? Water Resources Research. 2017;53(8):7029–54. 10.1002/2017WR020461

[46] Schieder NW, Walters DC, Kirwan ML. Massive Upland to Wetland Conversion Compensated for Historical Marsh Loss in Chesapeake Bay, USA Estuaries and Coasts. 2017 10.1007/s12237-017-0336-9

[47] Kirwan ML, Walters DC, Reay WG, Carr JA. Sea level driven marsh expansion in a coupled model of marsh erosion and migration. Geophysical Research Letters. 2016;43(9):4366–73. 10.1002/2016GL068507

[48] Stralberg D, Brennan M, Callaway JC, Wood JK, Schile LM, Jongsomjit D, et al Evaluating Tidal Marsh Sustainability in the Face of Sea-Level Rise: A Hybrid Modeling Approach Applied to San Francisco Bay. PLOS ONE. 2011;6(11):e27388 10.1371/journal.pone.0027388 22110638 PMC3217990

[49] Linhoss AC, Kiker G, Shirley M, Frank K. Sea-Level Rise, Inundation, and Marsh Migration: Simulating Impacts on Developed Lands and Environmental Systems. Journal of Coastal Research. 2014:36–46. 10.2112/JCOASTRES-D-13-00215.1

[50] Feagin RA, Martinez ML, Mendoza-Gonzalez G, Costanza R. Salt marsh zonal migration and ecosystem service change in response to global sea level rise: a case study from an urban region Ecology and Society. 2010.

[51] US Army Corps of Engineers. Interim survey report, Morgan City, Louisiana and vicinity. New Orleans, LA: US Army Engineer District, 1963 Serial no. 63 Contract No.: Serial no. 63.

[52] Lovelace JK. Storm-tide elevations produced by Hurricane Andrew along the Louisiana coast, August 25–27, 1992. Report. 1994 94–371.

[53] Resio D, Westerink J. Modeling the physics of storm surges. Physics Today. 2008;(9):33–8. citeulike-article-id:6354684.

[54] Siverd CG, Hagen SC, Bilskie MV, Braud DH, Peele RH, Twilley RR. Hydrodynamic storm surge model simplification via application of land to water isopleths in coastal Louisiana. Coastal Engineering. 2018;137:28–42. 10.1016/j.coastaleng.2018.03.006.

[55] Stark J, Oyen T, Meire P, Temmerman S. Observations of tidal and storm surge attenuation in a large tidal marsh. Limnology and Oceanography. 2015;60(4):1371–81. 10.1002/lno.10104

[56] O’Sullivan WT, Criss GA. Continuing Erosion in Southeastern Coastal Mississippi-Point aux Chenes Bay, West Grand Bay, Middle Bay, Grande Batture Islands: 1995–1997. Sixty-Second Annual Meeting of the Mississippi Academy of Sciences; February 26–27, 1998; Biloxi Mississippi1998.

[57] Peterson MS, Waggy GL, Woodrey MS. Grand Bay National Estuarine Research Reserve: An Ecological Characterization. Grand Bay National Estuarine Research Reserve, Moss Point, MS, 2007.

[58] Morton RA. Historical Changes in the Mississippi-Alabama Barrier-Island Chain and the Roles of Extreme Storms, Sea Level, and Human Activities. Journal of Coastal Research. 2008;24(6):1587–600.

[59] Gilmer B, Brenner J, Sheets J. Informing conservation planning using sea-level rise and storm surge impact estimates in the Grand Bay NERR/NWR area in Mississippi The Nature Conservancy, 2011.

[60] Eleuterius CK, Criss GA. Point aux Chenes: Past, Present, and Future Persepctive of Erosion. Ocean Springs, Mississippi: Physical Oceanography Section Gulf Coast Research Laboratory 1991.

[61] Clough JS, Polaczyk A. SLAMM Analysis of Grand Bay NERR and Environs. A report prepared for The Nature Conservancy. Waitsfield, VT: 2011.

[62] Resources MDoM. Mississippi’s Coastal Wetlands. Biloxi, MS: Coastal Preserves Program, 1999.

[63] Otvos EG. Barrier island evolution and history of migration, north central Gulf Coast Barrier Islands from the Gulf of St Lawrence to the Gulf of Mexico. 1979:291–319.

[64] Esri. World Imagery basemap. Sources: Esri, DigitalGlobe, GeoEye, i-cubed, USDA FSA, USGS, AEX, Getmapping, Aerogrid, IGN, IGP, swisstopo, and the GIS User Community; 2012. http://www.arcgis.com/home/item.html?id=86de95d4e0244cba80f0fa2c9403a7b2

[65] Miller-Way TL, Dardeau M, Crozier G. Weeks Bay National Estuarine Research Reserve: An Estuarine Profile and Bibliography. Dauphin Island Sea Lab, 1996 Contract No.: Technical Report 96–01.

[66] Weeks Bay National Estuarine Research Reserve. Weeks Bay National Estuarine Research Reserve Management Plan. Weeks Bay National Estuarine Research Reserve, 2007.

[67] Lu Z, McCormick BC, Faison C, April GC, Raney DC, Schroeder WW. Numerical Simulation of a Shallow Estuary—Weeks Bay, Alabama Estuarine and Coastal Modeling 1992 p. 418–29.

[68] Shirley L, Battaglia L. Assessing vegetation change in coastal landscapes of the northern Gulf of Mexico. Wetlands. 2006;26(4):1057–70. 10.1672/0277-5212(2006)26[1057:AVCICL]2.0.CO;2

[69] Morris JT, Sundberg K, Hopkinson CS. Salt marsh primary production and its responses to relative sea level and nutrients in estuaries at Plum Island, Massachusetts, and North Inlet, South Carolina, USA. Oceanography. 2013;26(3):78–84.

[70] Morris J, Hagen S, Medeiros S, Weishampel J, Edwards J, Alizad K, editors. Forecasting Current and Future Carbon Stocks in Gulf Coast Estuaries. AGU Fall Meeting Abstracts; 2013.

[71] Neubauer SC. Contributions of mineral and organic components to tidal freshwater marsh accretion. Estuarine, Coastal and Shelf Science. 2008;78(1):78–88. 10.1016/j.ecss.2007.11.011.

[72] Kirwan ML, Guntenspergen GR. Feedbacks between inundation, root production, and shoot growth in a rapidly submerging brackish marsh. Journal of Ecology. 2012;100(3):764–70. 10.1111/j.1365-2745.2012.01957.x

[73] van de Koppel J, Bouma TJ, Herman PMJ. The influence of local- and landscape-scale processes on spatial self-organization in estuarine ecosystems. The Journal of Experimental Biology. 2012;215(6):962. 22357589 10.1242/jeb.060467

[74] Turner RE, Swenson EM, Milan CS. Organic and inorganic contributions to vertical accretion in salt marsh sediments In: Weinstein M, Kreeger D, editors. Concepts and Controversies in Tidal Marsh Ecology. Boston: Kluwer Academic Publishers; 2000 p. 583–95.

[75] Mudd SM, Howell SM, Morris JT. Impact of dynamic feedbacks between sedimentation, sea-level rise, and biomass production on near-surface marsh stratigraphy and carbon accumulation. Estuarine, Coastal and Shelf Science. 2009;82(3):377–89. 10.1016/j.ecss.2009.01.028.

[76] Luettich RA, Westerink JJ. Formulation and numerical implementation of the 2D/3D ADCIRC finite element model version 44. XX: R. Luettich; 2004.

[77] Luettich R, Westerink J. ADCIRC: A parallel advanced circulation model for oceanic, coastal and estuarine waters; users manual for version 45.08. 2006.

[78] Bilskie MV, Hagen SC, Medeiros SC, Cox AT, Salisbury M, Coggin D. Data and numerical analysis of astronomic tides, wind-waves, and hurricane storm surge along the northern Gulf of Mexico. Journal of Geophysical Research: Oceans. 2016;121(5):3625–58. 10.1002/2015JC011400

[79] Medeiros SC, Hagen SC. Review of wetting and drying algorithms for numerical tidal flow models. International Journal for Numerical Methods in Fluids. 2013;71(4):473–87. 10.1002/fld.3668

[80] Arcement GJ, Schneider VR. Guide for selecting Manning’s roughness coefficients for natural channels and flood plains: US Government Printing Office Washington, DC, USA; 1989.

[81] Medeiros SC, Hagen SC, Weishampel JF. Comparison of floodplain surface roughness parameters derived from land cover data and field measurements. Journal of Hydrology. 2012;452–453:139–49. 10.1016/j.jhydrol.2012.05.043.

[82] Parris A, Bromirski P, Burkett V, Cayan D, Culver M, Hall J, et al. Global Sea Level Rise Scenarios for the US National Climate Assessment. 2012.

[83] Passeri DL, Hagen SC, Plant NG, Bilskie MV, Medeiros SC, Alizad K. Tidal hydrodynamics under future sea level rise and coastal morphology in the Northern Gulf of Mexico. Earth’s Future. 2016;4(5):159–76. 10.1002/2015EF000332

[84] Passeri DL, Hagen SC, Medeiros SC, Bilskie MV. Impacts of historic morphology and sea level rise on tidal hydrodynamics in a microtidal estuary (Grand Bay, Mississippi). Continental Shelf Research. 2015;111:150–8. 10.1016/j.csr.2015.08.001.

[85] Bilskie MV, Hagen SC, Alizad K, Medeiros SC, Passeri DL, Needham HF, et al Dynamic simulation and numerical analysis of hurricane storm surge under sea level rise with geomorphologic changes along the northern Gulf of Mexico. Earth’s Future. 2016;4(5):177–93. 10.1002/2015EF000347

[86] Bilskie MV, Coggin D, Hagen SC, Medeiros SC. Terrain-driven unstructured mesh development through semi-automatic vertical feature extraction. Advances in Water Resources. 2015;86, Part A:102–18. 10.1016/j.advwatres.2015.09.020.

[87] Bilskie MV, Hagen SC. Topographic accuracy assessment of bare earth lidar-derived unstructured meshes. Advances in Water Resources. 2013;52:165–77. 10.1016/j.advwatres.2012.09.003.

[88] Pitchford JL, Spurrier L, Barrett S. High resolution land cover map for the Grand Bay National Estuarine Research Reserve. Moss Point, Mississippi: Grand Bay National Estuarine Research Reserve, 2017.

[89] NWI. Mississippi coast updates to NWI based on imagery from 2006 and 2007 [Internet]. 2008. https://www.fws.gov/wetlands/Data/SupMapInf/R04Y08P10.pdf.

[90] National Oceanic and Atmospheric Administration, Center for Operational Oceanographic Products and Services. COOPS. Mean sea level trend at Dauphin Island, Alabama 2017. https://tidesandcurrents.noaa.gov/sltrends/sltrends_station.shtml?id=8735180.

[91] Cohen J. A Coefficient of Agreement for Nominal Scales. Educational and Psychological Measurement. 1960;20(1):37–46. 10.1177/001316446002000104

[92] Landis JR, Koch GG. The Measurement of Observer Agreement for Categorical Data. Biometrics. 1977;33(1):159–74. 10.2307/2529310 843571

[93] Monserud RA, Leemans R. Comparing global vegetation maps with the Kappa statistic. Ecological Modelling. 1992;62(4):275–93. 10.1016/0304-3800(92)90003-W.

[94] Jin S, Yang L, Danielson P, Homer C, Fry J, Xian G. A comprehensive change detection method for updating the National Land Cover Database to circa 2011. Remote Sensing of Environment. 2013;132:159–75. 10.1016/j.rse.2013.01.012.

[95] Esri. National Geographic World basemap. National Geographic, Esri, DeLorme, NAVTEQ, UNEP-WCMC, USGS, NASA, ESA, METI, NRCAN, GEBCO, NOAA, iPC 2012. http://www.arcgis.com/home/item.html?id=e35c5b72d6ac4928a8047ad95fec1618

[96] Dalrymple RW, Zaitlin BA, Boyd R. Estuarine facies models: conceptual basis and stratigraphic implications: perspective. Journal of Sedimentary Research. 1992;62(6).

[97] Crosby SC, Sax DF, Palmer ME, Booth HS, Deegan LA, Bertness MD, et al Salt marsh persistence is threatened by predicted sea-level rise. Estuarine, Coastal and Shelf Science. 2016;181:93–9. 10.1016/j.ecss.2016.08.018.

[98] Guerreiro M, Bustorff Fortunato A, Freire P, Rilo A, Taborda R, Freitas MC, et al Evolution of the hydrodynamics of the Tagus estuary (Portugal) in the 21st century. Revista de Gestão Costeira Integrada-Journal of Integrated Coastal Zone Management. 2015;15(1).

[99] Enwright NM, Griffith KT, Osland MJ. Barriers to and opportunities for landward migration of coastal wetlands with sea-level rise. Frontiers in Ecology and the Environment. 2016;14(6):307–16. 10.1002/fee.1282

[100] Passeri DL, Hagen SC, Medeiros SC, Bilskie MV, Alizad K, Wang D. The dynamic effects of sea level rise on low-gradient coastal landscapes: A review. Earth’s Future. 2015;3(6):159–81. 10.1002/2015EF000298

[101] Towns J, Cockerill T, Dahan M, Foster I, Gaither K, Grimshaw A, et al XSEDE: Accelerating Scientific Discovery. Computing in Science & Engineering. 2014;16(5):62–74. 10.1109/MCSE.2014.80.

