# Correction: Dynamic responses and implications to coastal wetlands and the surrounding regions under sea level rise

**DOI:** 10.1371/journal.pone.0210134

**Published:** 2018-12-28

**Authors:** The PLOS ONE Staff

There is an error in affiliation 1 for authors Karim Alizad and Matthew V. Bilskie. Affiliation 1 should be: Louisiana State University, Center for Coastal Resiliency, Baton Rouge, Louisiana, United States of America.

There is an error in affiliation 2 for author Scott C. Hagen. Affiliation 2 should be: Louisiana State University, Center for Coastal Resiliency, Center for Computation & Technology, Department of Civil, and Environmental Engineering, Baton Rouge, Louisiana, United States of America.

The publisher apologizes for the errors.
